# SLC7A2 deficiency promotes hepatocellular carcinoma progression by enhancing recruitment of myeloid-derived suppressors cells

**DOI:** 10.1038/s41419-021-03853-y

**Published:** 2021-06-02

**Authors:** Suhong Xia, Jingwen Wu, Wangdong Zhou, Mingyu Zhang, Kai Zhao, Jingmei Liu, Dean Tian, Jiazhi Liao

**Affiliations:** 1 Department of Gastroenterology, Tongji Hospital of Tongji Medical College, Huazhong University of Science and Technology, Wuhan, 430030 Hubei Province China; 2 Institute of Liver and Gastrointestinal Diseases, Tongji Hospital of Tongji Medical College, Huazhong University of Science and Technology, Wuhan, 430030 Hubei Province China

**Keywords:** Cancer microenvironment, Cancer immunotherapy, Metastasis

## Abstract

The main reason for poor prognosis in hepatocellular carcinoma (HCC) patients is high metastasis and recurrence. Cancer progression depends on a tumor-supportive microenvironment. Therefore, illustrating the mechanisms of tumor immunity in underlying HCC metastasis is essential. Here, we report a novel role of solute carrier family 7 member 2 (SLC7A2), a member of the solute carrier family, in HCC metastasis. The reduction of SLC7A2 was an independent and significant risk factor for the survival of HCC patients. Upregulation of SLC7A2 decreased HCC invasion and metastasis, whereas downregulation of SLC7A2 promoted HCC invasion and metastasis. We further found that deficient SLC7A2 medicated the upregulation of CXCL1 through PI3K/Akt/NF-kκB pathway to recruit myeloid-derived suppressor cells (MDSCs), exerting tumor immunosuppressive effect. Moreover, we found that G9a-mediated di-methylation of H3K9 (H3K9me2) silenced the expression of SLC7A2 to suppress HCC metastasis and immune escape. In conclusion, G9a-mediated silencing of SLC7A2 exerts unexpected functions in cancer metastasis by fostering a tumor-supportive microenvironment through CXCL1 secretion and MDSCs recruitment. Thus, SLC7A2 may provide new mechanistic insight into the cancer-promoting property of MDSCs.

## Introduction

Hepatocellular carcinoma (HCC) is the third leading cause of cancer-related deaths worldwide^1^. Although a growing number of strategies have been achieved in the prevention, diagnosis, and treatment of HCC, such as surgical resection and a series of drugs including multi-kinase and immune checkpoint inhibitors for HCC^2^. The U.S. Food and Drug Administration (FDA)-approved treatment of small molecule multi-kinase inhibitors, sorafenib, regorafenib for advanced HCC remains unsatisfactory^3–5^. Immune checkpoint inhibitors including nivolumab and pembrolizumab are emerging in the treatment of malignant tumors including HCC but only a fraction of HCC patients show a positive effect^6^. Thus, it is urgently warranted to explore the molecular mechanism underlying HCC metastasis and novel therapeutic strategies.

Emerging evidence suggests that immune evasion is one of the major hallmarks of cancer^7^. The tumor microenvironment promotes tumor malignant progression and limits the effectiveness of immunotherapy for solid tumors^8^. It is well recognized that HCC is usually preceded by liver damage and chronic inflammatory stimulation, and is accompanied by infiltration of many immune cells^9,10^. Among them, often accompanied by the activation of immune checkpoint signals such as programmed cell death receptor 1 (PD-1) and its ligand (PD-L1) or recruitment of myeloid-derived suppressors cells (MDSCs)^11^. MDSCs represent a heterogeneous immature immunosuppressive myeloid cell population that can obtain immune privilege by inhibiting the T cell functions to promote tumor progression^12^. In addition to T cells, MDSCs also suppress NK cells and dendritic cells but activate Tregs^13,14^. The interaction of chemokines and their cognate receptors plays an important role in regulating the recruitment and transport of MDSCs to tumor sites^15^. However, the reason for MDSCs recruitment caused by hepatic oncogenic signaling and its molecular mechanism remain to be solved.

Epigenetic plasticity and alterations play a key role in affecting tumor initiation and progression^16^. Such as DNA methylation, chromatin remodeling, and histone modification are highly associated with HCC proliferation and metastasis^17^. Among them, histone methylation plays a vital role in silencing gene expression. Especially, euchromatic histone lysine methyltransferase 2 (G9a, EHMT2) mainly catalyzes histone H3 lysine (H3K9) di-methylation and is upregulated expression in different types of tumors. In HCC, overexpression G9a is critically related to aggressive clinical prognosis, besides this involved in the mediating multiple cellular processes^18^. We little know that deregulation of G9a how to affect the abnormal epigenetic silencing in HCC. Therefore, it is necessary to study the functions and precise mechanism of G9a and downstream targeted genes.

Solute carrier family 7 member 2 (SLC7A2) is an important member of the cationic amino acid transporter (CAT) protein family^19^. SLC7A2 actively transports L-arginine (L-Arg) across the cell membrane and into the cytosol by y^+^ transporters^20^. The previous study reports that mice lacking SLC7A2 is accompanied by pro-tumorigenic M2 macrophage and exacerbates inflammation-associated colon tumorigenesis^21^. However, the expression and functional role of SLC7A2 in human HCC immunity remain largely unknown.

In this study, we aimed at the role of SLC7A2 in HCC metastasis and demonstrated that SLC7A2 knockdown promoted HCC metastasis by increasing MDSCs recruitment. This study might be a prospective therapeutic strategy for HCC.

## Results

### SLC7A2 is significantly downregulated in HCC tissues and deficient SLC7A2 indicates a poor prognosis

**Fig. 1:**
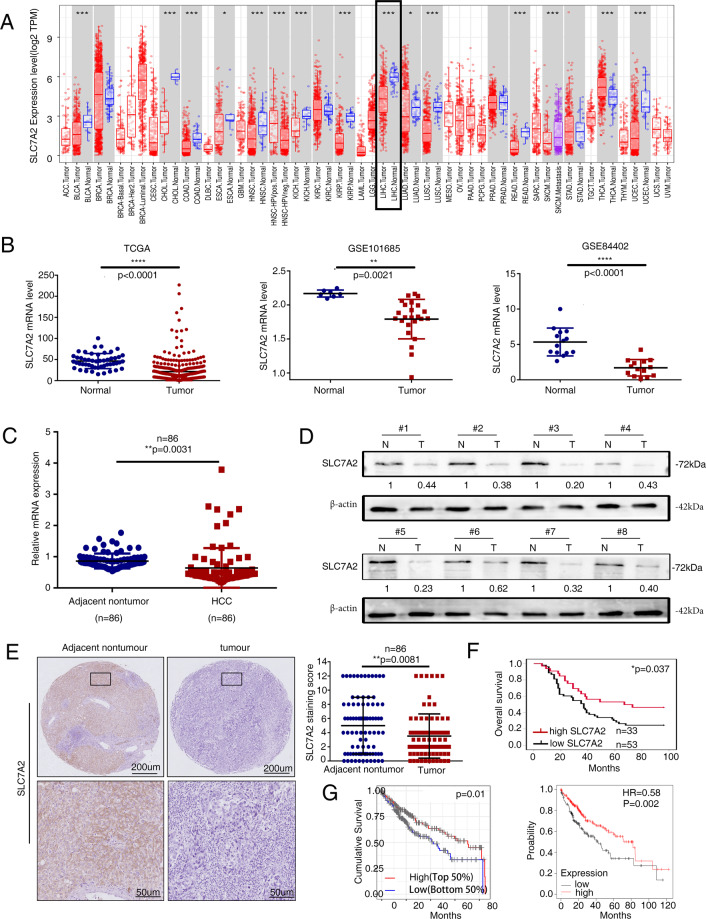
SLC7A2 is significantly downregulated in HCC tissues and deficient SLC7A2 indicates a poor prognosis. **A** Expression of SLC7A2 in diverse cancer types from the TIMER website. **B** Bioinformatics analysis of SLC7A2 mRNA expression on the TCGA and the GEO database. (TCGA, GSE101685, GSE84402 contains 50, 7, 14 normal tissues and 374, 24, 14 HCC tissues, respectively). **C** Relative SLC7A2 mRNA expression in 86 paired HCC and adjacent nontumorous tissues. **D** Western blot analysis of SLC7A2 expression in eight pairs of human HCC samples and paired adjacent nontumor tissues. **E** Representative image of the IHC staining for SLC7A2. IHC scores of SLC7A2 in the HCC cohort. Scale bars, 200 µm (upper), 50 µm (lower). **F** Kaplan–Meier analysis of the association of SLC7A2 expression and overall survival in the HCC cohort. **G** Kaplan–Meier analysis of overall survival was compared according to the expression levels of SLC7A2 in HCC tissues from the TIMER and Kaplan–Meier Plotter website. All the data were shown as the mean ± s.d. *P < 0.05, **P < 0.01, ***P < 0.001, ****P < 0.0001.

We first analyzed the expression of SLC7A2 in multiple cancers using the TIMER website and confirmed the overexpression of SLC7A2 in normal tissues than HCC tissues (Fig. 1A). In addition, data from The Cancer Genome Atlas (TCGA) and two GEO datasets showed that elevated expression of SLC7A2 in liver normal tissues (Fig. 1B). We sequentially evaluated SLC7A2 expression in 86 paired HCC tissues. The mRNA expression of SLC7A2 was significantly decreased in HCC tissues than in adjacent nontumor tissues (Fig. 1C). A similar result was observed in western blotting (Fig. 1D). Bioinformatics analysis for SLC7A2 expression between HCCs and nontumor HCCs demonstrated that SLC7A2 was significantly downregulated in multiple tumors (Supplementary Fig. 1A, B). We subsequently analyzed the protein expression and clinical significance of SLC7A2 with a tissue array of 86 HCC patient samples using immunohistochemical (IHC) staining. The SLC7A2 protein levels were significantly decreased in HCC tissues compared with that in adjacent nontumor tissues (Fig. 1E). Deficiency SLC7A2 in HCC patients had shorter overall survival than patients with elevated expression (Fig. 1F). Our results were supported by other database data on the survival of SLC7A2 (Fig. 1G). Negative SLC7A2 expression was significantly increased tumor size and higher tumor-nodule-metastasis (TNM) stage (Table 1). Together, these data indicated that SLC7A2 was a prospective prognostic biomarker in HCC patients.

**Table 1:** Correlation between SLC7A2 expression and clinicopathological characteristics in HCC patients.

Clinicopathological variables	Tumor SLC7A2 expression		*P* Value
	Negative (*n* = 53)	Positive (*n* = 33)	
Age(years)			
<55	28	21	0.224
≥55	25	12	
Sex			
Female	8	11	0.063
Male	45	22	
Serum AFP			
≤20 ng/ml	7	8	0.245
>20 ng/ml	46	25	
Cirrrhosis			
Absent	6	8	0.139
Present	47	25	
Child-pugh score			
Class A	43	29	0.552
Class B	10	4	
Tumor number			
Single	43	22	0.196
Multiple	10	11	
Maximal tumor size			
≤5 cm	24	29	<0.001*
>5 cm	29	4	
Tumor encapsulation			
Absent	28	9	0.026
Present	25	24	
Microvascular invasion			
Absent	20	19	0.080
Present	33	14	
TNM stage			
I–II	31	29	0.004*
III	22	4	

*Indicates statistical significance.

### Knock-down SLC7A2 promotes HCC proliferation, invasion, and metastasis in vitro

**Fig. 2:**
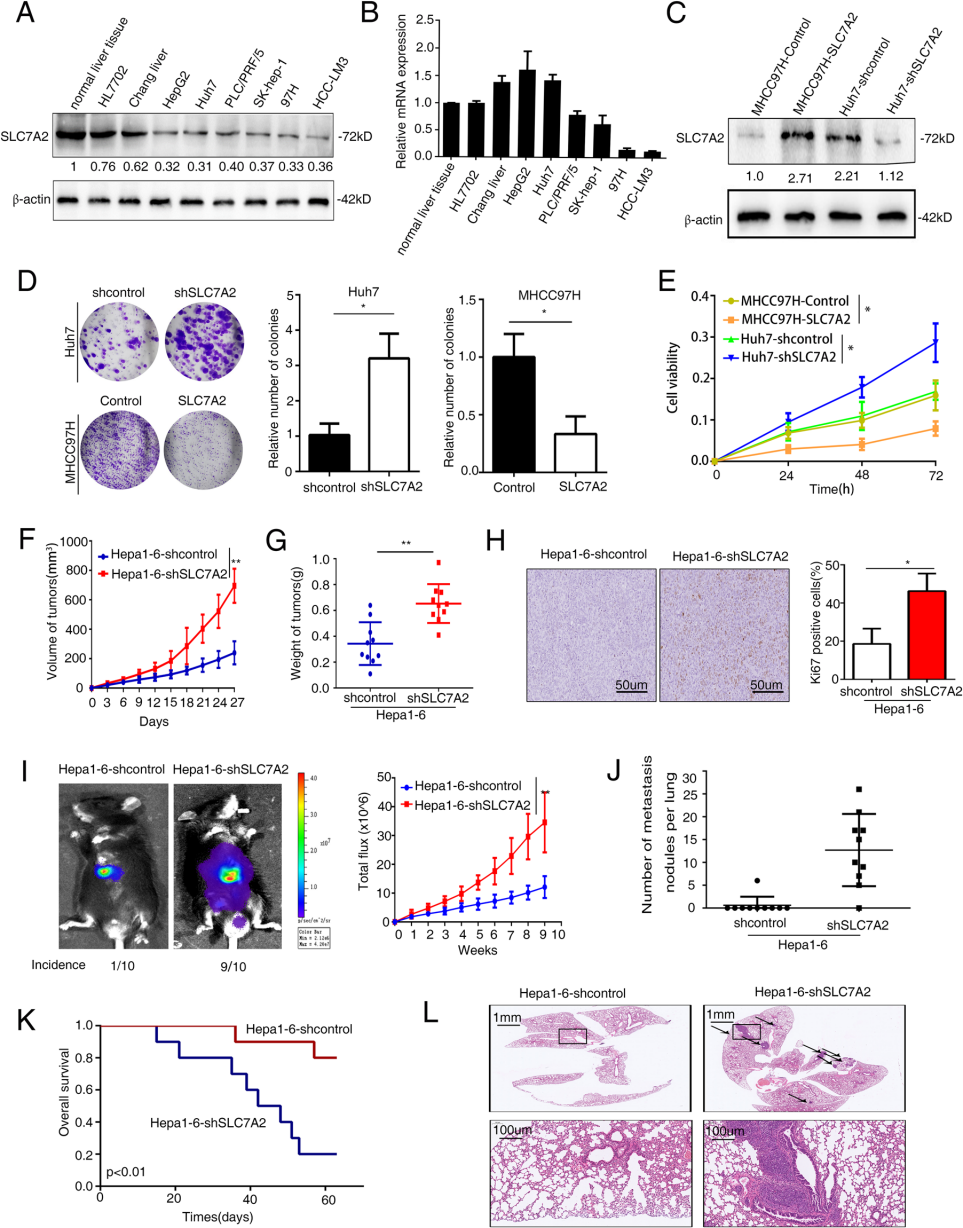
SLC7A2 disruption facilitates HCC proliferation, invasion, and metastasis in vitro and vivo. **A**, **B** Relative mRNA and protein expression of SLC7A2 in normal liver tissue and HCC cell lines. **C** Western blotting analyzed performed the expression of SLC7A2 in Huh7 and MHCC97H cells after lentivirus transfection. **D**, **E** Deficient SLC7A2 promoted HCC cell proliferation in vitro. **D** Effects of SLC7A2 on HCC cell colony formation. **E** The effects of SLC7A2 on HCC cell proliferation were measured by a CCK-8 assay. The data are shown as the mean ± SD from the at least three independent experiments; *P < 0.05. **F**–**L** Deficient SLC7A2 promoted HCC cell growth and migration in vivo. **F** Growth curves and **G** weight curves of tumors in C57BL/6 (*n* = 10 mice per group) in subcutaneously model. **H** IHC staining for Ki67 in the indicated tumors. **I** Representative Bioluminescent images and total photon flux were shown. **J** Incidence of lung metastasis and the number of metastatic nodules in the different group. **K** Overall survival time in C57BL/6 mice. **L** Representative images of H&E stained-lung tissues from the different. The scale bars represent 1 mm (upper panel) and 100 μm (lower panel). The data are shown as the mean ± SD; *P < 0.05, **P < 0.01.

We then detected SLC7A2 expression in HCC cell lines and found that SLC7A2 level was higher in HCC cells with low metastatic capability (Fig. 2A, B). Huh7 and MHCC97H cells were selected to establish stable cell lines, Huh7-shSLC7A2 and MHCC97H-SLC7A2, with lentivirus infection (Fig. 2C). Colony formation assays indicated that deficient SLC7A2 significantly increased HCC cell proliferation (Fig. 2D), which was consistent with the results of the cell counting kit 8 (CCK8) assays (Fig. 2E). In addition, we found that SLC7A2 level was higher in Hepa1-6 cells than in H22 cells (Supplementary Fig. 2A). We next used Hepa1-6 cells to establish stable cell lines, Hepa1-6-shSLC7A2 with lentivirus infection (Supplementary Fig. 2B). Cell counting kit 8 assays showed that knock-down SLC7A2 in mice HCC cells markedly enlarged HCC cell proliferation (Supplementary Fig. 2C). Furthermore, overexpression SLC7A2 decreased the migration and invasion of MHCC97H, while knockdown SLC7A2 in Huh7 cells increased the invasion and migration (Supplementary Fig. 2D). We also found the same result that high level of SLC7A2 suppressed mice HCC cells migration and invasion but deficient SLC7A2 had greatly improved the ability of migration and invasion (Supplementary Fig. 2E). We have done more work to understand the mechanism of SLC7a2 regulating tumor growth and metastasis in vitro. There have been reported that cell apoptosis and EMT (epithelial–mesenchymal transitions) play a key role in proliferation and metastasis^22–24^. We tested the effect of SLC7A2 on apoptosis by flow cytometry. The results demonstrated that SLC7A2 silence significantly decreased the percentage of apoptotic cells, and SLC7A2 overexpression increased the percentage of apoptotic cells (Supplementary Fig. 2F). Furthermore, western blot analysis verified that the protein expression levels of Twist1, Vimentin, N-cadherin, and MMP9 increased while E-cadherin decreased in the SLC7A2 knockdown group but achieved the opposite effect in the SLC7A2 overexpression group (Supplementary Fig. 2G). These data showed that SLC7A2 knockdown inhibited cell apoptosis to promote tumor growth and survival, and increased EMT to regulate metastasis in vitro. Based on these findings, SLC7A2 served as a tumor suppressor gene that suppresses HCC cell growth and metastasis in vitro.

### SLC7A2 disruption facilitates HCC proliferation, invasion, and metastasis in vivo by mediating HCC immune escape

Because cationic amino acid transporter 2 (CAT2) is required for myeloid-derived suppressor cell-mediated of T cell immunity^25^. However, SLC7A2 function in regulating tumor responses to tumor immunity has not been explored. We wanted to explore the SLC7A2 function in regulating tumor responses to tumor immunity, so we performed subcutaneous model with SLC7A2 knockdown Hepa1-6 cells in immune-competent C57BL/6 and immunodeficient mice. A subcutaneous mouse model was used in immunodeficient mice and the data indicated that tumor growth was not affected by SLC7A2 deficiency in immunodeficient mice (Supplementary Fig. 3A). In addition, the IHC staining for Ki67 showed that SLC7A2 in the proliferation of tumor cells was no difference (Supplementary Fig. 3B). Interestingly, an immunocompetent subcutaneous HCC mouse model in C57BL/6 was served as tumorigenesis and the results showed that SLC7A2 overexpression significantly suppressed tumor growth (Fig. 2F, G). IHC staining for Ki67 in the tumor cells suggested that suppressed SLC7A2 in the proliferation of tumor cells has largely increased (Fig. 2H). Next, we demonstrated the representative images of the IHC staining about immunity cells (Supplementary Fig. 4A). IHC staining showed that knock-down SLC7A2 tumor cells were positively CD11b but negatively CD8. These findings indicated that SLC7A2 disruption-mediated tumor growth attenuation may depend on tumor immunity.

Furthermore, knockdown SLC7A2 promoted intrahepatic and lung metastasis and number of metastatic lung nodules, resulting in extended overall survival time in Hepa1-6-shSLC7A2 than its controls with intrahepatic tumor implantation experiment (Fig. 2I–L). These data demonstrated the dual character of SLC7A2 disruption, which served as a tumor suppressor gene that suppressed HCC cell growth and metastasis while improving HCC progression by promoting HCC immune escape.

### Low SLC7A2 induces CXCL1 secretion and MDSC infiltration

**Fig. 3:**
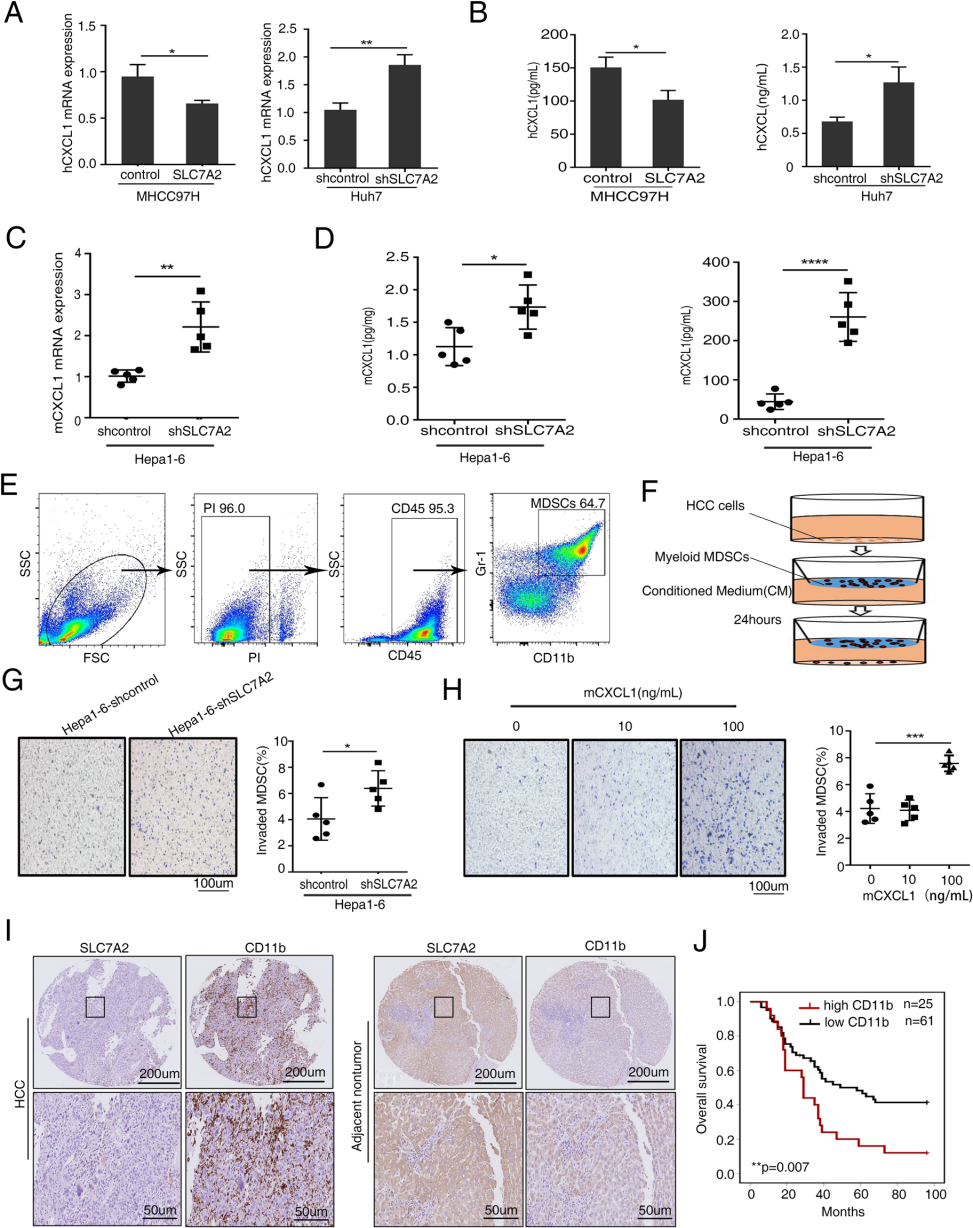
Low SLC7A2 induces CXCL1 secretion and MDSC infiltration. **A**, **B** Levels of CXCL1 in human HCC cells by qRT-RCR and ELISA. The data are shown as the mean ± SD from the at least three independent experiments; *P < 0.05, **P < 0.01. **C** Levels of CXCL1 by qRT-RCR in Hepa1-6-shSLC7A2 tumor-bearing mice tumors (*n* = 5). The data are shown as the mean ± SD from the at least three independent experiments; **P < 0.01. **D** ELISA of CXCL1 in subcutaneous tumors and in blood from tumor-bearing mice. (*n* = 5). The data are shown as the mean ± SD from the at least three independent experiments; *P < 0.05, ****P < 0.0001. **E** Labeled CD11b^+^Gr-1^+^ cells myeloid MDSCs from Hepa1-6 bearing mice were determined by fluorescence-activated cell sorted (FACS). **F** Schematic diagram about the migration of MDSCs. **G** Conditioned media (CM) from Hepa1-6-shSLC7A2 and their controls clones were placed in the lower chambers. Freshly Myeloid MDSCs were put in the upper chambers and invasion for 24 h. Total numbers were counted. The data are shown as the mean ± SD from the at least three independent experiments; *P < 0.05. **H** Pre-treated with mCXCL1 (0, 10,100 ng/mL, 24 h), the chemotaxis of mouse MDSCs is shown. The data are shown as the mean ± SD from the at least three independent experiments; ****P < 0.0001. **I** Representative IHC staining images for SLC7A2 and CD11b in human HCC tissues. Scale bars, 200 µm (upper), 50 µm (lower). **J** Kaplan–Meier analysis of the associations between MDSCs infiltration and overall survival in the HCC cohort. The data are shown as the mean ± SD; **P < 0.01.

Tumor from deficient SLC7A2 mice exhibited significantly increases levels of the multiple proinflammatory cytokines and chemokines, which is accompanied by pro-tumorigenic M2 macrophage activation^21^. MDSCs have been reported to differentiate into tumor-associated macrophages (TAMs), especially the M-MDSCs^26^. To figure out the functions of TAMs, we examined the indices of TAMs in the tumor site. Flow cytometric analysis showed that TAMs had no significant difference in hepa1-6-shcontrol and hepa1-6-shSLC7A2 subcutaneous tumors in C57BL/6 mice (Supplementary Fig. 5A). The myeloid-derived suppressor cells (MDSCs), tumor‐associated macrophages (TAMs) and Treg cells are major suppressive immune cells in tumor sites, and their dominant roles are obstructing T‐cell activities and facilitating tumor survival and progression^27^. The results showed that the TAMs could not play a critical role in SLC7A2-mediated tumors. Moreover, SLC7A2 contributes to the transport of L-Arginine in MDSC and is an important regulator of MDSC suppressive function^25^. Given that SLC7A2 serves as a regulator MDSCs which contributes to immune responses and cytokines and chemokines, so we intended to figure out whether SLC7A2 affects MDSCs function resulting in tumor immunity and cytokines and chemokines to promote HCC progression. We used a chemokines and receptors RT^2^ profiler PCR Array to find prospective chemokines between Huh7-shSLC7A2 and Huh7-shcontrol cells. Decreased SLC7A2 level induced the expression of multiple liver cancer-related genes, CXCL1 was strongly induced by deficient SLC7A2 expression (Supplementary Table S1). Notably, CXCL1 is the key chemokine in the tumor microenvironment by recruiting MDSCs^28^. Considering the vital role of the CXCL1 in immunoregulation, we decided to analyze the CXCL1 expression in HCC cells. The result that CXCL1 was induced by SLC7A2 knockdown was established by Quantitative real-time polymerase chain reaction (qRT-PCR) and enzyme-linked immunosorbent assay (ELISA) (Fig. 3A, B). Next, we showed the subcutaneous mouse tumors with SLC7A2 knockdown in immune-competent C57BL/6 mice. In this model, the CXCL1 level in decreased SLC7A2 tumors was higher than its control group (Fig. 3C). Meanwhile, CXCL1 concentrations in the blood and tumors were higher in mice with downregulated SLC7A2 tumors (Fig. 3D).

Next, we analyzed the chemotactic ability of CXCL1 on MDSCs. We detected the proportion of myeloid MDSCs from Hepa1-6 bearing mice. The flow cytometry showed that the proportion of myeloid MDSCs from Hepa1-6 bearing mice was up to 64.7% (Fig. 3E). The chemotaxis assay indicated that the MDSCs were mainly attracted by higher CXCL1 (Fig. 3F, G). The migration of MDSCs was augmented by CXCL1 in a dose-dependent manner (Fig. 3H).

We further assessed the correlation between SLC7A2 and MDSC infiltration in the HCC cohort. We demonstrated the representative images of the IHC staining in Fig. 3I. Overexpression of MDSC infiltration was mainly correlated with microvascular invasion (Supplementary Table S2). Patients with overexpression of MDSC infiltration demonstrated a shorter overall survival than those with downregulated MDSC infiltration (Fig. 3J). These findings demonstrated that deficiency of SLC7A2 upregulates CXCL1 and induces MDSC chemotaxis, meanwhile, MDSC infiltration may indicate a poorer prognosis.

### Depletion of MDSC using anti-Gr-1 inhibits low SLC7A2-mediated HCC metastasis

**Fig. 4:**
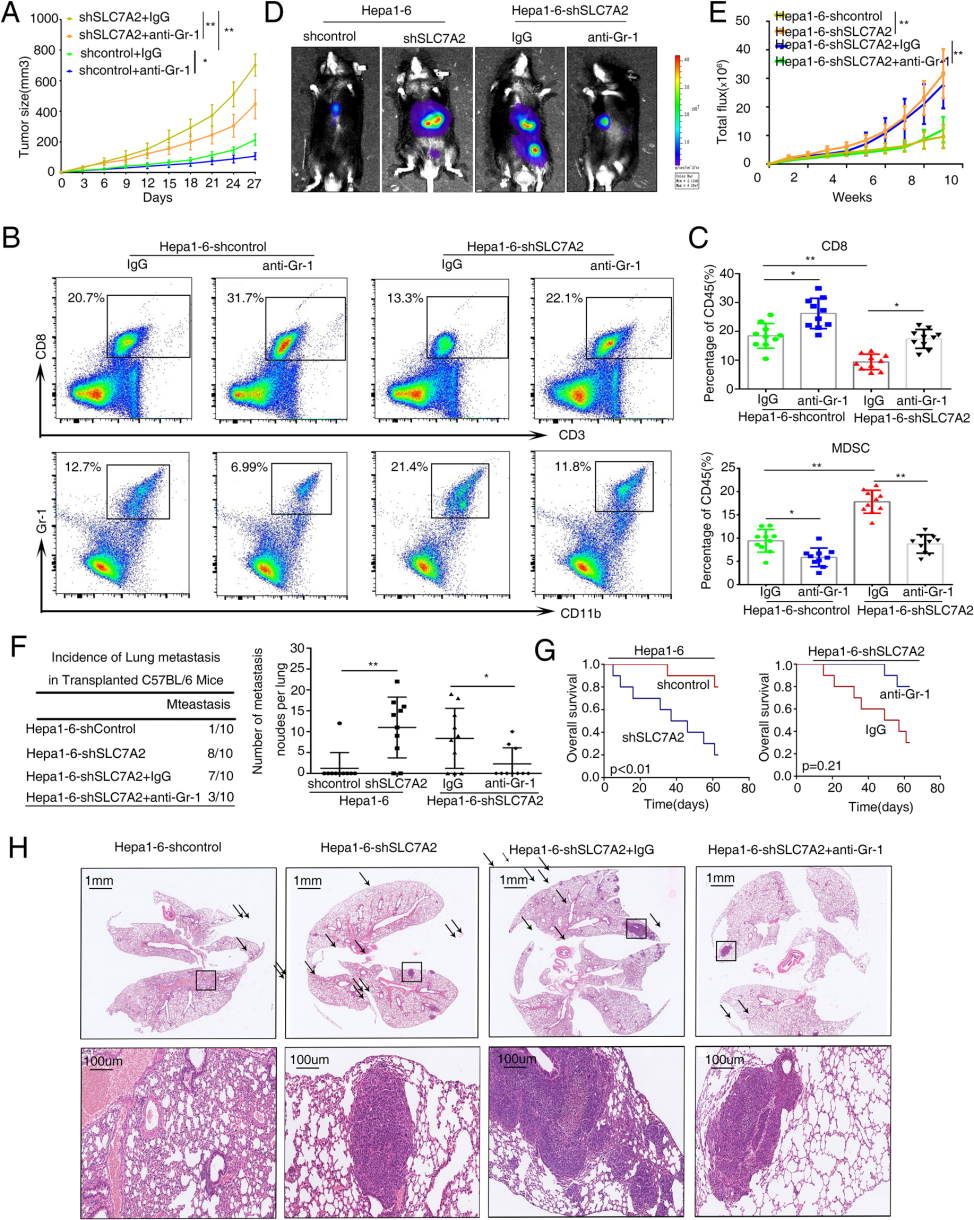
Depletion of MDSC using anti-Gr-1 inhibits low SLC7A2 mediated HCC metastasis. **A** Tumor growth in mice subcutaneously injected with Hepa1-6-shcontrol or Hepa1-6-shSLC7A2 cells, treated with anti-Gr-1 antibody (400 μg/body) or IgG twice a week from day 1 after tumor inoculation. *n* = 10. **B** Flow cytometric images of subcutaneous Hepa1-6-shcontrol or Hepa1-6-shSLC7A2 cells treated with anti-Gr-1 antibody or IgG at day 27. CD8^+^ T cells (upper) and MDSCs (lower). **C** Flow cytometric analyses the ratio of CD8^+^ T cells and MDSCs between groups at day 27 respectively, *n* = 10. The data are shown as the mean ± SD from the at least three independent experiments; **P < 0.01. **D**–**H** In vivo assays shown that MDSCs depletion could suppress loss of SLC7A2-mediated HCC metastasis. **D** The representative Bioluminescence images were shown in the different groups, treated with anti-Gr-1 antibody or IgG in the C57BL/6 injected with the indicated cells in the liver, *n* = 10. **E** The Bioluminescence intensity in the tumors at the indicated time point was presented as the total photon flux. **F** Incidence of lung metastasis and the number of metastatic lung nodules in lung in the C57BL/6 mice. *n* = 10. **G** Overall survival of the mice in each group. **H** Representative H&E-stained lung metastatic nodules. The scale bars represent 1 mm (upper panel) and 100 μm (lower panel). The data are shown as the mean ± SD; *P < 0.05 and **P < 0.01.

We have so far understood that SLC7A2 deficiency induces MDSCs chemotaxis by upregulating the overexpression of CXCL1. MDSCs could play a role in antitumor immunity by suppressing CD8^+^ T cells to promote HCC immune escape^29^. We next exhibited tumor-bearing mouse models to analyze the in vivo function of MDSC suppression by anti-Gr-1 antibody. MDSCs depletion using anti-Gr-1 antibody inhibited tumor growth and it was more effective for larger tumors size (Fig. 4A). We further used the flow cytometric analysis found that the anti-Gr-1 antibody could deplete MDSCs in its control groups and the effect is more obvious in the Hepa1-6-shSLC7A2 group with larger tumor size. Additionally, we found that tumor-infiltrating MDSCs decreased, and the proportion of CD8^+^ cells increased in anti-Gr-1 antibody-treated tumors (Fig. 4B, C). Myeloid-derived suppressor cells (MDSCs) develop through the myelopoiesis pathway of bone marrow and spleen, which are heterogeneous bone marrow cells and exert immunosuppressive effects^30^. The percentage of spleen MDSCs cells were distinctly decreased whereas the percentage of spleen CD8^+^ T cells was significantly increased with the treatment of anti-Gr-1 antibody (Supplementary Fig. 5B). However, the proportion of MDSCs in the tumor was significantly higher than that in the spleen. Briefly, these data showed that anti-Gr-1 antibodies decreased the percentages of MDSCs cells in tumors and spleen and the MDSCs in tumors may play a decisive role in tumor immunosuppression.

Furthermore, the in vivo metastatic assay demonstrated that knockdown of SLC7A2 increased the incidence of lung metastasis and the number of metastatic nodules and had a longer overall survival of the Hepa1-6-shSLC7A2 group (Fig. 4D–H). In the Hepa1-6-shSLC7A2 mice groups, treatment with anti-Gr-1 antibody resulted in a lower rate incidence of lung metastasis, and fewer number of metastatic nodules and longer overall survival than without anti-Gr-1 antibody treatment (Fig. 4D–H). These results suggested the anti-Gr-1 antibody decreased MDSC immunosuppression and thus inhibited low SLC7A2-mediated HCC metastasis.

### Low SLC7A2 upregulates CXCL1 expression by transactivating P65 expression

**Fig. 5:**
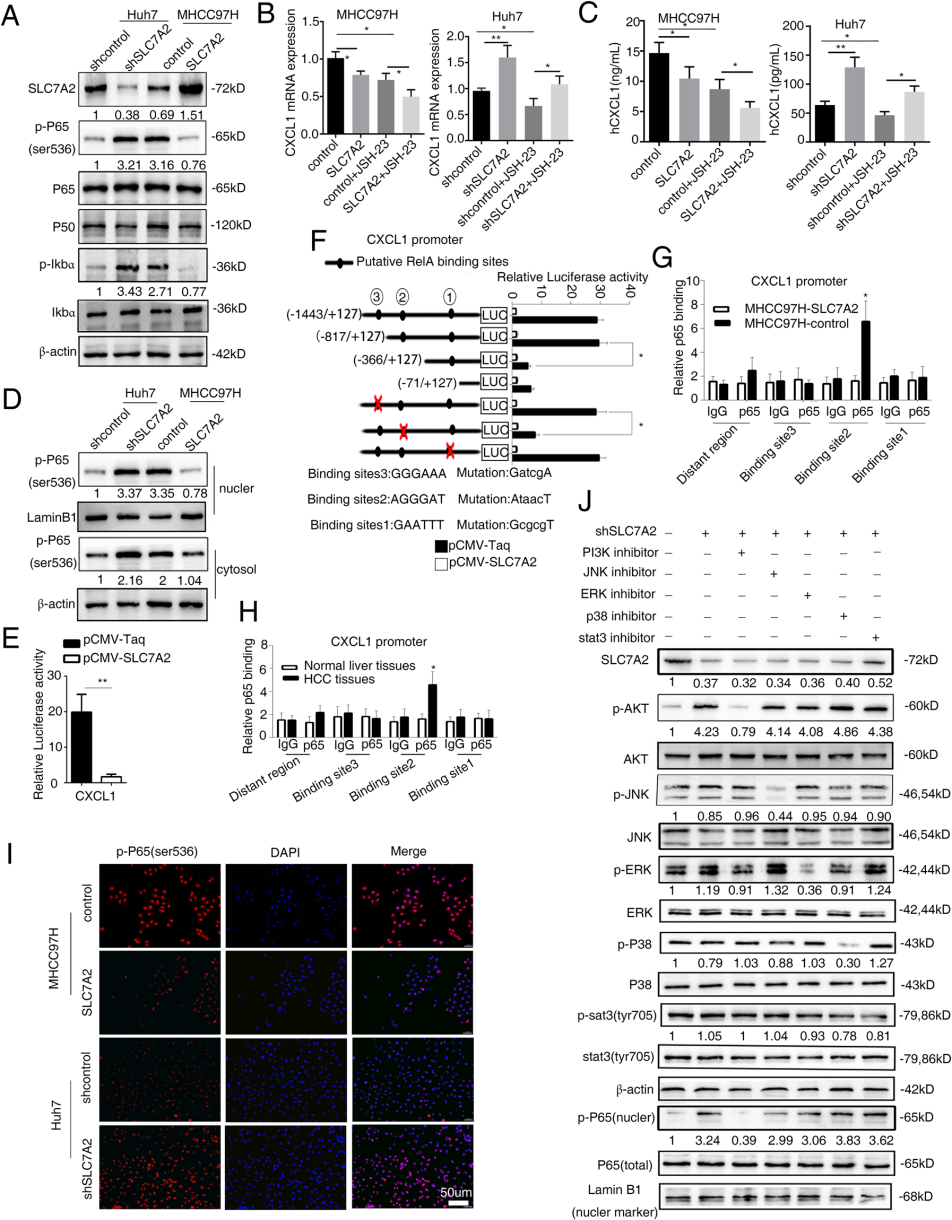
Low SLC7A2 upregulates CXCL1 expression by transactivating P65 expression. **A** Western Blotting the SLC7A2, phosphorylated P65 at ser^536^ (p-P65^Ser536^), P65, P50, p-IKBα, IKBα, β-actin was used as control. **B** qRT-PCR was used to detect that relative mRNA expression of CXCL1 in the indicated cells with treatment with NF-kκB inhibitor, JSH-23 (10 μm, 24 h). The data are shown as the mean ± SD from the at least three independent experiments; *P < 0.05, **P < 0.01. **C** ELISA was used to detect that relative expression of CXCL1 in the indicated cells with treatment with NF-κB inhibitor, JSH-23 (10 μm, 24 h). The data are shown as the mean ± SD from the at least three independent experiments; *P < 0.05, **P < 0.01. **D** The expression of phosphorylated P65 was analyzed in the nuclear and cytoplasmic fractions of Huh7-shSLC7A2 cells and MHCC97H-SLC7A2 cells. The laminB1 and β-actin were as control respectively. **E** Luciferase reporter assay was showed in the indicated co-transfected with pCMV-SLC7A2 and the CXCL1 promoter-luciferase construct. The data are shown as the mean ± SD from the at least three independent experiments; **P < 0.01. **F** Truncated and mutated CXCL1 promoter constructs were co-transfected with PCMV-SLC7A2, and the relative luciferase activity was confirmed. The data are shown as the mean ± SD from the at least three independent experiments; *P < 0.05. **G** ChIP assay confirmed the direct binding of P65 to the CXCL1 promoter in HCC cells. The data are shown as the mean ± SD from the at least three independent experiments; *P < 0.05. **H** ChIP assay confirmed the direct binding of P65 to the CXCL1 promoter in human HCC tissues. The data are shown as the mean ± SD from the at least three independent experiments; *P < 0.05. **I** Immunofluorescence of p-P65^Ser536^ of indicated HCC cells. **J** Huh7-shSLC7A2 cells treated with inhibitors of PI3K, JNK, ERK, p38, STAT3, and then western blotting was used to detect the expression of SLC7A2 and p-P65^Ser536^ in the nuclear as well as the total and phosphorylate expression of AKT, ERK, JNK, p38, STAT3.

It has been previously reported that NF-κB pathway activates CXCL1 gene transcription^28^. We hypothesized that P65 would be activated during low SLC7A2-induced metastasis, which would enhance CXCL1 expression. To verify this hypothesis, we tested the protein level of NF-kκB pathway in both Huh7 and MHCC97H cells by western blot analysis. Phosphorylation level of P65 which an essential role in the canonical NF-kB pathway, was markedly increased in knockdown SLC7A2 cells such as Huh7-shSLC7A2 and MHCC97H-control, and was decreased in the Huh7-shcontrol and MHCC97H-SLC7A2 (Fig. 5A). However, the level of phosphorylation IKBα, as well as the P50 and P65, did not change. The results suggested that deficient SLC7A2 might induce phosphorylation of P65 in HCC cells. We were next convinced that NF-kκB promoted CXCL1 gene transcription. When treatment with the NF-kκB inhibitor, JSH-23, CXCL1 expression decreased in Huh7 and MHCC97 cells by qRT-PCR and ELISA (Fig. 5B, C). In addition, we extracted and analyzed the cytoplasmic and nuclear proteins, the results indicated that P65 phosphorylation significantly increased in the nucleus of SLC7A2 knockdown HCC cells (Fig. 5D).

Luciferase reporter assay showed that upregulation of SLC7A2 suppressed the CXCL1 promoter activity (Fig. 5E). To figure out how SLC7A2 deficiency regulated CXCL1, the promoter sequences of CXCL1 were analyzed and three putative CXCL1 binding motifs were found in the CXCL1 promoter. Then a series of luciferase reporter plasmids with truncated or mutated CXCL1 promoter sequences were constructed. Several deletions suggested that the region between −1443 to +127 bp was vital to the SLC7A2-mediated expression of luciferase reporter (Fig. 5F). In addition, chromatin immunoprecipitation (ChIP) assays indicated that P65 is directly bound to the CXCL1 promoter in MHCC97H-control cell lines and human HCC tissues (Fig. 5G, H). These data indicated that SLC7A2 deficiency might increase the binding activity of P65 to the CXCL1 promoter.

Moreover, we demonstrated that immunofluorescence staining for phosphorylated P65, and the result suggested phosphorylated P65 fluorescence was strong and mainly localized to the nucleus in MHCC97-control and Huh7-shSLC7A2 cells (Fig. 5I). Loss of SLC7A2 reduces the transport of L-Arg and thus inhibits the activation of cGMP-dependent protein kinase II (PKG II) enhanced PI3K/Akt signaling pathway^31^. In addition, it is reported that SLC7A11 from the same family as SLC7A2 can regulate and affect the protein expression of PI3K and Akt signaling pathways^32^. Simultaneously, the PI3K/Akt signaling pathway regulating the expression of P65 has been mentioned in several tumors^33^. We supposed that knockdown SLC7A2 was responsible for the P65 expression by PI3K/Akt signaling pathway. Besides that, p38 kinases, c-Jun-N-terminal kinase (JNK), ERK, and STAT3 could induce activation of NF-kκB^34^. To certify these hypotheses, Huh7 cells were treated with PI3K, ERK, JNK, P38, STAT3 inhibitors. Pretreatment of cells with PI3K inhibitor significantly reduced knockdown SLC7A2-mediated phosphorylated P65 in the nucleus overexpression. However, pretreating cells with ERK, JNK, P38, STAT3 inhibitors did not notably affect SLC7A2-induced P65 expression (Fig. 5J). Collectively, these results indicated that deficient SLC7A2 upregulated CXCL1 expression by phosphorylating P65 and promoting P65 nuclear translocation through PI3K/Akt pathway.

### SB265610, an effective CXCR2 inhibitor, suppresses CXCL1 recruitment for MDSC and low SLC7A2-mediated HCC metastasis

**Fig. 6:**
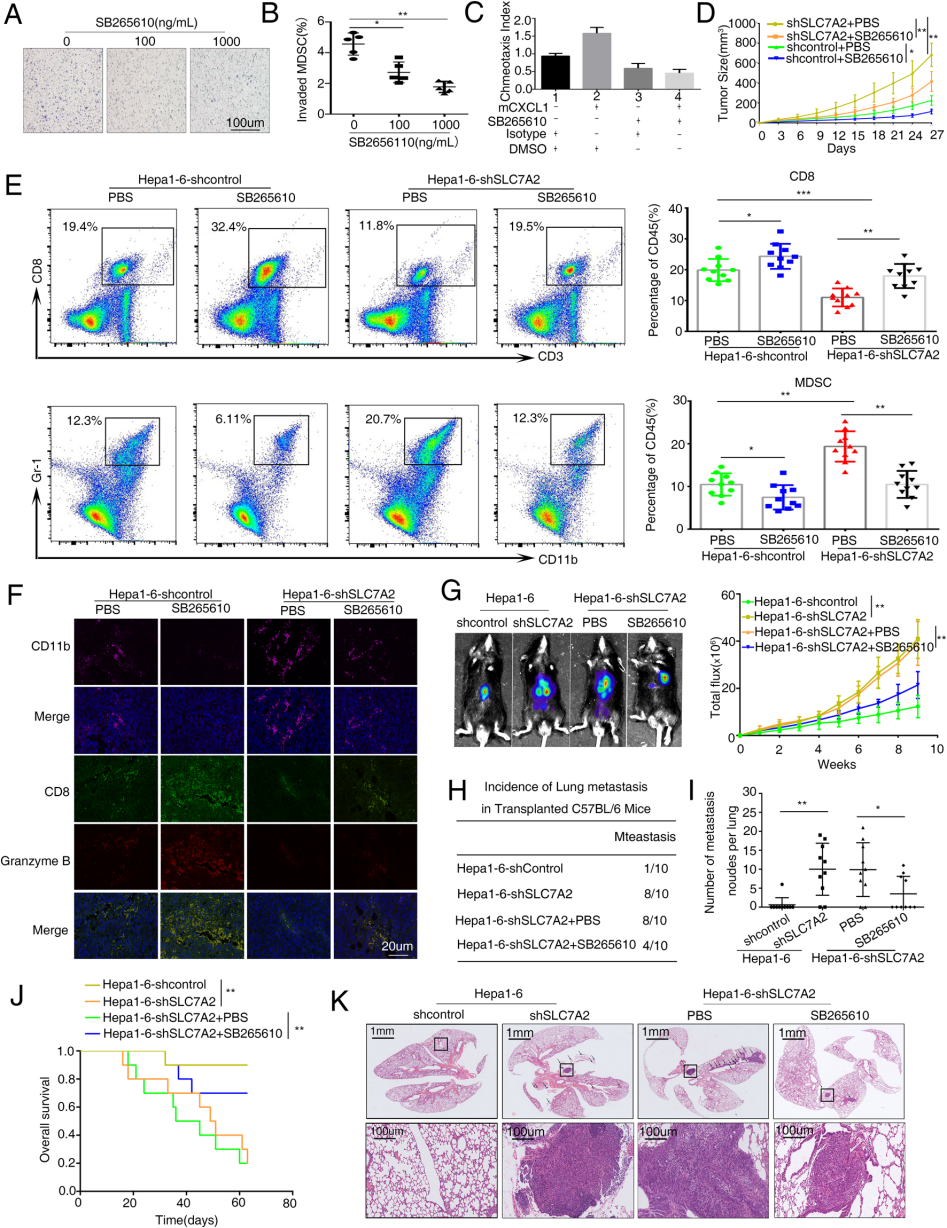
Treatment of CXCR2 antagonist suppresses low SLC7A2-mediated HCC metastasis. **A**, **B** Conditioned media (CM) from Hepa1-6 clones was placed in the lower chambers with different concentration of SB265610 (0, 100,1000 ng/mL, 24 h). Freshly Myeloid MDSCs were put in the upper chambers and invasion for 24 h. Total numbers were counted. The scale bars represent 100 μm. The data are shown as the mean ± SD from the at least three independent experiments; *P < 0.05, **P < 0.01. **C** Pre-treated with mCXCL1 (10 ng/mL, 24 h) and SB265610 (100 ng/mL, 24 h), the chemotaxis index of mouse MDSCs is shown. The data are shown as the mean ± SD from the at least three independent experiments; *P < 0.05. **D** Tumor growth in mice subcutaneously injected with Hepa1-6-shcontrol or Hepa1-6-shSLC7A2 cells, treated with CXCR2 antagonist (SB265610: 2 mg/kg body weight) or PBS six times a week from day 1 after tumor inoculation. *n* = 10. **E** Flow cytometric images of subcutaneous Hepa1-6-shcontrol or Hepa1-6-shSLC7A2 cells treated with SB265610 or PBS at day 27. CD8^+^ T cells (upper) and MDSCs (lower). Flow cytometric analyses the ratio of CD8^+^ T cells and MDSCs between groups at day 27 respectively, *n* = 10. The data are shown as the mean ± SD from the at least three independent experiments; *P < 0.05, **P < 0.01, and ***P < 0.001. **F** Immunofluorescent staining of the CD11b, CD8, and granzyme B protein expression patterns in mice tumor cells. **G**–**K** In vivo assays shown that the treatment of SB265610 can block knock-down SLC7A2-mediated HCC metastasis. **G** The representative Bioluminescence images were shown in the different groups, treated with SB265610 or PBS in the C57BL/6 injected with the indicated cells in the liver, *n* = 10. The Bioluminescence intensity in the tumors at the indicated time point was presented as the total photon flux. **H** Incidence of lung metastasis **I** the number of metastatic lung nodules in lung in the C57BL/6 mice. *n* = 10. **J** Overall survival of the mice in each group. **K** Representative H&E-stained lung metastatic nodules. The scale bars represent 1 mm (upper panel) and 100 μm (lower panel). Images shown are representative of at least three independent experiments. *P < 0.05, **P < 0.01.

The MDSC is recruited by CXCL1 through the expression of the cognate receptor CXCR2, thus treatment with CXCR2 antagonist (SB265610) inhibits MDSC chemotaxis induced by chemokines^28^. Our previous researches had shown that CXCL1 promoted HCC metastasis by recruiting MDSC. Thus, we wanted to know whether the recruitment blockage of MDSC could disturb deficient SLC7A2-mediated HCC metastasis. The chemotaxis assay showed that migration of MDSCs was reduced with SB265610 in a dose-dependent manner (Fig. 6A, B). SB265610 was treated and CXCL1 was added at the same time, which could appropriately promote the migration of MDSCs (Fig. 6C). Tumor-bearing mouse models exhibited that in vivo treatment using CXCR2 antagonist (SB265610) inhibited tumor growth, and Hepa1-6-shSLC7A2 tumor-bearing mice group was more significant (Fig. 6D). We further used flow cytometric analysis found that MDSCs decreased but CD8^+^ T cells increased in tumors and Hepa1-6-shSLC7A2 group had better effects (Fig. 6E). Consistently, the MDSCs were decreased but the CD8^+^ T cells and granzyme B increased in the tumor tissues of mice given CXCR2 antagonist (Fig. 6F). As expected, in vivo metastatic assay suggested that SB265610 treatment decreased the incidence of lung metastasis and the number of metastasis nodules and had a longer overall survival of the Hepa1-6-shSLC7A2 group (Fig. 6G–K). These results suggested the CXCR2 antagonist reversed immunosuppression through the inhibition of MDSC migration to the tumor, thereby blocking low SLC7A2 mediated HCC metastasis.

### G9a inhibitor, UNC0642, suppresses SLC7A2-mediated HCC immune escape, invasion, and metastasis

**Fig. 7:**
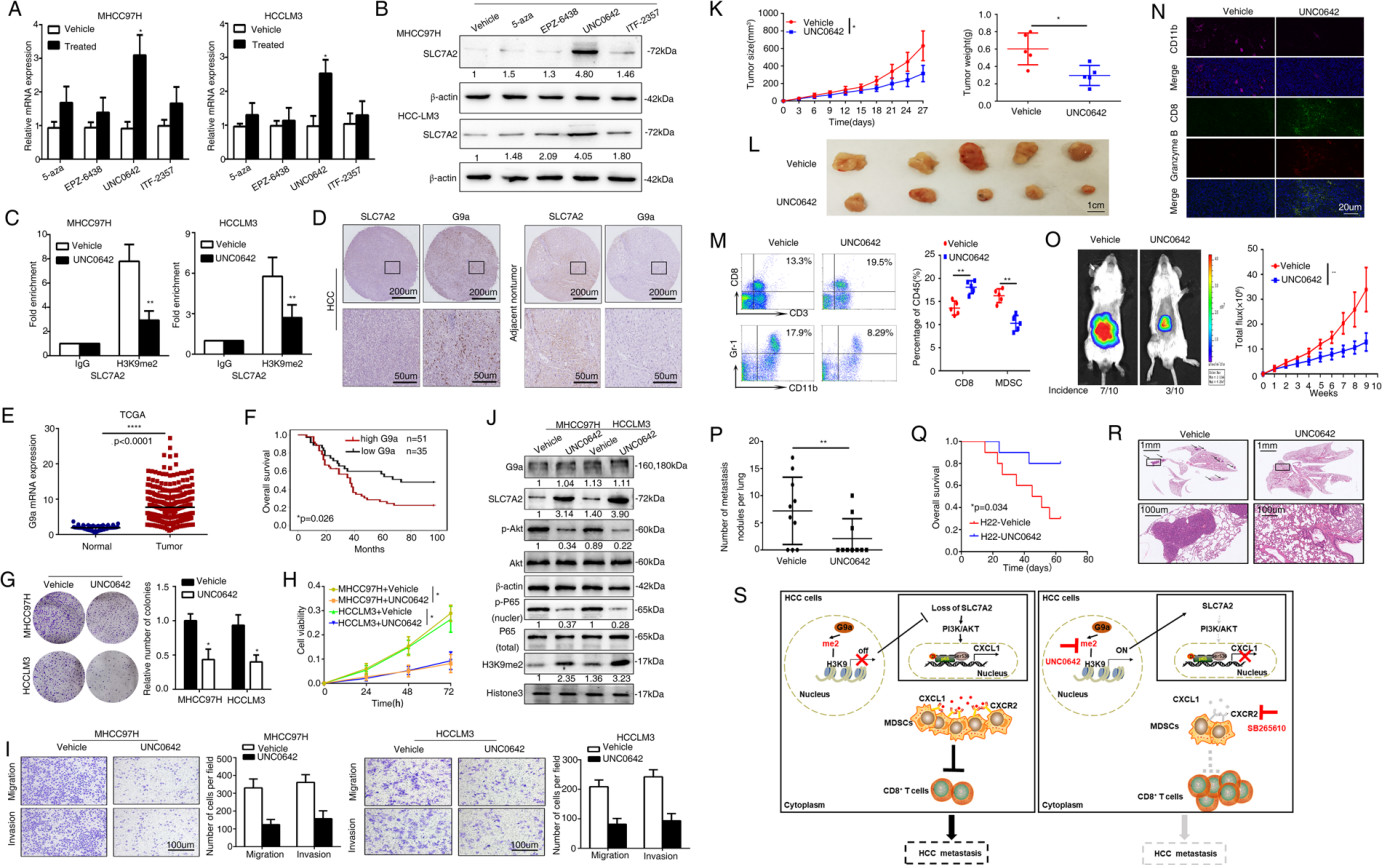
G9a inhibitor, UNC0642, suppresses SLC7A2-mediated HCC immune escape, invasion, and metastasis. **A**, **B** MHCC97H and HCCLM3 HCC cells treated with 5-aza (2 μm, 3 days), EPZ-6438 (1 μm, 3 days), UNC0642 (5 μm, 2 days), ITF-2357 (1 μm, 3 days) then qRT-RCR and western blotting was used to test the expression of SLC7A2. The data are shown as the mean ± SD from the at least three independent experiments; *P < 0.05. **C** ChIP-quantitative real-time PCR analysis to detect H3K9me2 association with SLC7A2 gene after treatment of G9a inhibitor UNC0642. The data are shown as the mean ± SD from the at least three independent experiments; **P < 0.01. **D** Representative IHC staining images for SLC7A2 and G9a in human HCC tissues. Scale bars, 200 µm (upper), 50 µm (lower). **E** Bioinformatics analysis of G9a mRNA expression on the TCGA database in human HCC tissues. **F** Kaplan–Meier analysis of the associations between G9a expression and overall survival in the HCC cohort. **G**–**I** Treatment of UNC0642 promoted HCC cell proliferation and metastasis in vitro. **G** Usage of UNC0642 on HCC cell colony formation. **H** The effects of UNC0642 on HCC cell proliferation were measured by a CCK-8 assay. **I** Transwell assay shown the abilities of migration and invasion. The scale bar represents 100 μm. **J** Western blotting was used to detect the G9a, SLC7A2, and its downstream target genes expression in MHCC97H and HCCLM3 cells treated with UNC0642. The data are shown as the mean ± SD from the at least three independent experiments; *P < 0.05. **K**, **L** Tumor growth in mice subcutaneously injected with H22-control or H22-SLC7A2 cells, treated with UNC0642 daily by intraperitoneal injection at 5 mg/kg. The tumor volume was monitored every 3 days. *n* = 5. **M** Flow cytometric images of subcutaneous H22-control or H22-SLC7A2 cells treated with UNC0642 or its control at day 27. CD8^+^ T cells (upper) and MDSCs (lower). Flow cytometric analyses the ratio of CD8^+^ T cells and MDSCs between groups at day 27, respectively, *n* = 5. The data are shown as the mean ± SD from the at least three independent experiments; **P < 0.01. **N** Immunofluorescent staining of the CD11b, CD8, and granzyme B protein expression patterns in mice tumor cells. Scale bars, 20 µm. **O**–**R** In vivo assays shown that the treatment of UNC0642 can block knock-down SLC7A2-mediated HCC metastasis. **O** The representative Bioluminescence images were shown in the different groups, treated with UNC0642 or DMSO in the BALB/C injected with the indicated cells in the liver and Incidence of lung metastasis, *n* = 10. The Bioluminescence intensity in the tumors at the indicated time point was presented as the total photon flux. **P** The number of metastatic lung nodules in lung in the BALB/C mice. *n* = 10. **Q** Overall survival of the mice in each group. **R** Representative H&E-stained lung metastatic nodules. The scale bars represent 1 mm (upper panel) and 100 μm (lower panel). *P < 0.05, **P < 0.01. **S** A schematic diagram of the SLC7A2 signaling in HCC immune evasion. SLC7A2 deficiency upregulated CXCL1 expression through PI3K/Akt/NF-κB pathway. CXCL1 promoted HCC growth and metastasis through recruiting MDSCs to the tumors. Neutralizing or suppressing MDSC infiltration abolished deficient SLC7A2-mediated HCC growth and metastasis. Usage of G9a inhibitor (UNC0642) suppressed loss of SLC7A2-meidated HCC metastasis.

DNA methylation and histone deacetylation contribute to aberrant gene silencing^18^. Therefore, we guessed whether DNA and histones alterations can cause the decline of SLC7A2. Multiple bioinformatics sites analyzed the correlation between epigenetics and SLC7A2 and suggested that G9a (also known as EHMT2) showed a significant negative correlation with SLC7A2 expression (Supplementary Fig. 6A–C). Using inhibitors of DNA methylation and histone modification, but only the G9a inhibitor reversed SLC7A2 upregulation (Fig. 7A, B). To verify the interaction of G9a with its target gene, chromatin immunoprecipitation (ChIP) assays confirmed that G9a interacted with SLC7A2 through H3K9me2 (Fig. 7C). We next demonstrated the G9a was mainly located in the nucleus (Fig. 7D). The data from TCGA showed that the expression of G9a was higher in tumors than in normal tissues (Fig. 7e). Patients with elevated G9a demonstrated shorter overall survival than those with downregulated G9a in the cohort (Fig. 7F). Upregulated G9a was mainly correlated with tumor number (Supplementary Table S3).

Then we performed colony formation assays, CCK-8 assays and transwell assays showed that HCC cells treated with G9a inhibitor UNC0642 greatly decreased the cell proliferation, migratory and invasive abilities in vitro (Fig. 7G–I). Moreover, due to treatment of UNC0642, not only the H3K9me2 level but SLC7A2 and its downstream genes were substantially diminished (Fig. 7J). We analyzed the SLC7A2 expression in H22 cells and next used H22 cells to establish stable cell lines, H22-SLC7A2 with lentivirus infection (Supplementary Fig. 2A, B). Subcutaneous tumor models confirmed that in vivo using G9a inhibitor (UNC0642) relieved tumor growth in BALB/C mice (Fig. 7K, L). To further explore the effects of G9a inhibitors on tumor immunity, we used flow cytometric analysis found that MDSCs decreased but CD8^+^ T cells increased in BALB/C mice treated with G9a inhibitor (Fig. 7M). Sequentially, the CD8^+^ T cells and granzyme B were expanded but the MDSCs decreased in the mice given G9a inhibitor (Fig. 7N). Concurrently, in vivo metastatic assay suggested that UNC0642 treatment decreased the incidence of lung metastasis and the number of metastasis nodules and had longer overall survival (Fig. 7O–R). These data exhibited the G9a inhibitor overturn immune escape through increasing the expression SLC7A2, hence upregulated the level of SLC7A2 preventing HCC metastasis.

## Discussion

The tumor microenvironment favors the growth and expansion of cancer cells^35^. It is increasingly clear that the tumor microenvironment contributes to invasion, metastasis, and chronic inflammation^36^. Although there are many immunotherapies targeted the tumor microenvironment, the effectiveness is limited^37^. In this study, we intended to clear the function of the tumor-supportive microenvironment in HCC metastasis.

At present, studies have found that SLC7A2 plays critical roles in ovarian cancer and breast cancer^38,39^. Loss of SLC7A2 augments inflammation-associated colon tumorigenesis^21^. An increasing number of studies show that SLC7A2 is related to tumor progression and inflammation responses, but its importance and detailed molecular mechanisms remain unknown. In our work, we found that SLC7A2 was deficient in HCC tissues. Loss of SLC7A2 elevated HCC cell proliferation, invasion and metastasis in vitro and in vivo, however, upregulation of SLC7A2 inhibited HCC cell metastasis. Furthermore, SLC7A2 played a role in suppressing tumors in HCC and enhanced tumor immune surveillance in the tumor microenvironment.

Myeloid-derived suppressor cells (MDSCs) are immature myeloid cells with immunosuppressive activity and are abundantly found in HCC^10^. The generation and accumulation of MDSCs mainly play the role of inhibiting CD8^+^T cells. Due to MDSCs vital role in obstructing immune responses, MDSCs are an effective strategic obstacle immunotherapies^40^. There have many chemokines and their receptors are important for MDSCs recruitment to tumor sites^41–43^. In our studies, we showed that MDSCs accumulated and increased but CD8^+^ decreased in HCC tumors. In addition, our data showed that deficient SLC7A2 drove MDSC recruitment by upregulation of CXCL1 through PI3K/Akt/NF-kκB pathway (Fig. 7S).

An anti-Gr-1 neutralizing antibody can effectively deplete MDSCs^44^. Thus, we found that using anti-Gr-1 neutralizing antibody could deplete MDSCs and suppress low SLC7A2-mediated HCC metastasis in vivo metastatic assay. Therefore, we knew that MDSC removal was likely to be a promising strategy for tumor immunotherapy. To design a pharmacological strategy against the recruitment of MDSCs via the CXCL1-CXCR2 axis, we concentrated on the CXCR2 antagonist (SB265610) confirmed to inhibit the recruitment of MDSCs^28,41^. Our findings presented that the usage of CXCR2 antagonist suppressed deficient SLC7A2-mediated HCC metastasis by restraining the recruitment of MDSCs. However, the specific role and effect of MDSCs derived from shSLC7A2 mediated tumors in tumor immunosuppression remain to be further studied.

There are many reports show that histone modifications regulate protein levels. G9a can affect tumor growth and invasion by regulating H3K9me2 activity^45^. However, the expression and function of G9a in human HCC remain unclear. In this study, we found that G9a played a significant role in promoting HCC progression. We treated the HCC cells with G9a inhibitors (UNC0642) and observed that UNC0642 significantly impeded cell proliferation and invasion. Notably, the tumor of growth and metastasis and even recruitment of MDSCs in HCC tumors were inhibited by the usage of UNC0642 in vivo. These data further supported G9a-mediated loss of SLC7A2 expression promoted MDSCs infiltration and induced tumor immune evasion.

In conclusion, we showed that deficient SLC7A2 was general in the majority of HCC patients and was an inducer of MDSC recruitment. Deficient SLC7A2 drove MDSC recruitment by upregulation of expression CXCL1 through PI3K/Akt/NF-kκB pathway, thus avoiding immune surveillance and promoting tumor progression. Neutralizing or suppressing MDSC infiltration abolished deficient SLC7A2-mediated HCC growth and metastasis. The treatment of G9a inhibitor significantly suppressed SLC7A2-mediated HCC metastasis. Collectively, these findings provided the important mechanism of SLC7A2-mediated tumor immune evasion through attracting MDSCs and put forth blockage of MDSCs recruitment as a prospective immunotherapy strategy for the treatment of SLC7A2 deficiency HCC metastasis.

## Materials and methods

### HCC specimens

A total of 86 adult patients with HCC underwent curative resection between 2009 and 2012 at the Tongji Hospital of Tongji Medical College (Wuhan, China). This study was approved by the Ethics Committee of Tongji Medical College. All HCC specimens were provided informed consent that was obtained in compliance with the guidelines of the Declaration of Helsinki. The clinicopathological characteristics of these patients were shown in Table 1.

### Construction of tissue microarrays and immunohistochemistry

HCC samples and the corresponding adjacent liver tissues were performed to construct a tissue microarray (Shanghai Biochip Co., Ltd. Shanghai, China). Immunohistochemistry was performed on 4-μm-thick. After baking at 60 °C for an hour, the tissue sections were deparaffinized with xylene and dehydrated through gradient ethanol immersion. Then the endogenous peroxidase activity was blocked with 3% (vol/vol) hydrogen peroxide. The tissue sections were incubated with the primary antibody in a moist chamber at 4 °C overnight. Then, the peroxidase-conjugated second antibody (Santa Cruz) was used to incubate the sections for 30 min at room temperature. The reaction product was observed with diaminobenzidine for 2 min. Images were used by a light microscope (Olympus, Japan) equipped with a DP70 digital camera.

IHC analyses were performed by two independent observers who were blinded to the clinical outcome. The percentage of positive cells was evaluated on a scale of 0–4: 0 (negative), 1 (1–25%), 2 (26–50%), 3 (51–75%), or 4 (76–100%). The immunostaining intensity was scored on a scale of 0–3: 0 (negative), 1 (weak), 2 (medium) or 3 (strong). Final immuno-activity scores were calculated by multiplying the above two scores, with final scores ranging from 0 to 12. Each case was ultimately considered “negative” if the final score was <4, and “positive” if the final score was ≥4.

The tissue microarray was stained for SLC7A2 (Abcam, ab140831, 1:200), CD11b (Cell signaling technology, #49420, 1:200), G9a (Abcam, ab133482 1:100) expression.

The mice tumor tissues were stained for SLC7A2 (Abcam, ab140831, 1:200), CD11b (Abcam, ab133357, 1:200) expression, CD8 (Cell Signaling Technology, #98941, 1:200), Ki67 (Abcam, ab15580, 1:200).

### Cell culture

Immortalized liver cell lines (HL-7702) and Chang liver were purchased from the Institute of Biochemistry and Cell Biology, Chinese Academy of Science, China. Human HCC cells (HepG2, Huh-7, PLC/PRF/5, MHCC97H, HCCLM3, and SK-hep-1) and mice HCC cells (H22, Hepa1-6) were purchased from the American Type Culture Collection. Cells were cultured in Dulbecco’s modified eagle medium (DMEM) and Roswell Park Memorial Institute (RPMI 1640) at 37 °C in a 5% CO_2_ incubator. The medium was supplemented with 10% FBS.

### Construction of lentivirus and stable cell lines

Lentiviral vectors encoding shRNAs were generated using PLKO.1-puro (Hanbio, Shanghai, China) and designated as human and mouse LV-shSLC7A2 and LV-shcontrol. “LV-shcontrol” is a non-target shRNA control. The shRNA sequences can be found in Supplementary Table S5. Lentiviral vectors encoding the human SLC7A2 genes and mouse SLC7A2 genes were constructed in PLKO.1-puro (Hanbio, Shanghai, China) and designated as LV-SLC7A2. An empty vector was used as the negative control and was designated as LV-control. Concentrated lentivirus was transfected into the HCC cells with a multiplicity of infection (MOI) ranging from 20 to 50 in the presence of polybrene (5 µg/ml). Seventy-two hours after infection, HCC cells were selected for 2 weeks using 2.5 µg/ml puromycin (OriGene). Selected pools of knockdown and overexpressing cells were used for the following experiments.

### Reagent

ERK inhibitor U0126, JNK inhibitor SP600125, p38 inhibitor SB203580, PI3K inhibitor LY294002, STAT3 inhibitor WP1066, NF-kB inhibitor JSH-23 were purchased from MedChemExpress (Monmouth Junction, NJ, USA). 5-aza, EPZ-6438, UNC0642, ITF-2345 were from Selleck (Houston, TX, USA). All the reagents were used according to the manufacturer’s instructions.

### Flow cytometry

For murine samples, mice with tumors were killed according to the institutional ethical guidelines and femurs, tibias, and tumors were collected. Signal cell of suspension of tumor was obtained by using the Tumor Dissociation Kit (Miltenyi Biotech, Bergish Gladbach, Germany, 130-096-730) according to the manufacturer’s instructions. Antibodies (BD Pharmingen, San Diego, CA, USA) used are listed: anti-mouse-CD3e (BD Pharmingen, 566494), anti-mouse-CD45 (BD Pharmingen, 553079), anti-mouse-CD8e (BD Pharmingen, 553035), anti-mouse-CD11b (BD Pharmingen, 550993), anti-mouse-Ly-6G/Ly-6C (BD Pharmingen, 553129), Anti-mouse-F4/80 (BD Pharmingen, 565410). For cell apoptosis assays, apoptosis cells were measured by a FITC Annexin V Apoptosis Detection Kit I (BD Pharmingen, San Diego, CA, USA) according to the manufacturer’s protocol. Data were acquired using FACS Calibur (BD Biosciences), and analyzed using Cell Quest Pro software (BD Biosciences)^46^.

### In vivo metastatic model and bioluminescent imaging

All animal procedures were carried out in accordance with the Guide for the Care and Use of Laboratory Animals and standards articulated in the Animal Research: Reporting of In Vivo Experiments. All animal experiments were approved by the Committee on the Tongji Hospital of Tongji Medical College, Huazhong University of Science and Technology. All animals were randomly grouped. C57BL/6 mice and BALB/C mice (male, 5 weeks old) were housed and cared according to the institutional guidelines for animal care. For in vivo metastasis assay, 2 × 10^6^ cells were resuspended in 50 μL PBS/Matrigel mixture. Under anesthesia, mice were orthotopically inoculated in the left hepatic lobe with the indicated cells through an 8-mm transverse incision in the upper abdomen (*n* = 10 mice/group). For intervention in C57BL/6 mice, the anti-Gr-1 antibody or IgG was treatment was initiated 1 day after tumor cell inoculation and was administered intraperitoneally twice a week (400 μg per body, Bioxcell, αGr-1, clone RB6-8C5)^47^. The CXCR2 antagonist or PBS treatment was initiated 1 day after tumor cell inoculation and was administered intraperitoneally six times a week (SB265610: 2 mg/kg body weight; R&D systems)^48^. For BALB/C mice, G9a inhibitor was administrated 7 days after tumor cell inoculation via intraperitoneal injection daily at 5 mg/kg (UNC0642, SelleckChem, #S7230)^18^. The Bioluminescent images were captured using a Lago X optical imaging system Imaging System (SI Imaging). At the 9 weeks, the mice were sacrificed and the lungs were collected for histological examination.

### In vivo tumor growth in the subcutaneous model

All animal experiments were approved by the Committee on the Tongji Hospital of Tongji Medical College, Huazhong University of Science and Technology. All animals were randomly grouped. An in vivo tumorigenesis subcutaneous model was established in 5-weeks-old male NOD/SCID mice, 5-weeks-old male C57BL/6 mice and BALB/C mice. Suspended treated cells were subcutaneously injected into the flank of each mouse (10 mice per group, 5 × 10^6^ cells in 100 μl of PBS per mouse). The mice were weighed and the tumor size was measured using a vernier caliper. The tumor volume was calculated using the following equation: maximum tumor diameter (L) × diameter at a right angle to that axis (W)^2^/2.

For intervention in C57BL/6 mice, the anti-Gr-1 antibody or IgG was treatment was initiated 1 day after tumor cell inoculation and was administered intraperitoneally twice a week (400 μg per body, Bioxcell, αGr-1, clone RB6-8C5)^49^. The CXCR2 antagonist or PBS treatment was initiated 1 day after tumor cell inoculation and was administered intraperitoneally six times a week (SB265610: 2 mg/kg body weight; R&D systems)^48^. Suspended treated cells were subcutaneously injected into the flank of each mouse (1 × 10^7^ cells in 100 μl of PBS /Matrigel mixture per mouse, ten in each group). The tumor size was measured using vernier calipers. For BALB/C mice, G9a inhibitor was administrated 7 days after tumor implantation via intraperitoneal injection daily at 5 mg/kg (UNC0642, SelleckChem, #S7230)^18^. After 27 days, the mice were killed according to the institutional ethical guidelines. After the mice were killed, the relevant tests were used in flow cytometry.

### Nuclear and cytoplasmic protein extraction

Nuclear and cytoplasmic protein extraction was analyzed using a Nuclear and Cytoplasmic Protein Extraction Kit (Beyotime, Shanghai, China) according to the manufacturer’s instructions.

### Plasmid construction

Plasmid construction was performed according to standard procedures as outlined. The primers are presented in Supplementary Table S4. For example, the CXCL1 promoter construct, (−1443/+127) CXCL1, was generated from human genomic DNA. This construct corresponds to the sequence from −1443 to +127 (relative to the transcriptional start site) of the 5′-flanking regions of the human CXCL1 gene. It was generated with forward and reverse primers incorporating KpnI and MluI sites at the 5′ and 3′-ends, respectively. The polymerase chain reaction (PCR) product was cloned into the KpnI and MluI sites of the pGL3-Basic vector (Promega, Madison, WI). The 5′-flanking deletion constructs of the CXCL1 promoter, (−817/+127) CXCL1, (−366/+127) CXCL1 and (−71/+127) CXCL1 were similarly generated using the (−1443/+127) CXCL1 construct as the template. The P65 binding sites in the CXCL1 promoter were mutated using the QuikChange II Site-Directed Mutagenesis Kit (Stratagene). The constructs were confirmed by DNA sequencing. Other promoter constructs were cloned in the same manner.

### Chemotaxis assays

In vitro migration of murine MDSCs was evaluated in 24-well plates with transwell polycarbonate-permeable supports (8.0 μm; Costar Corning, Cambridge, MA, USA). MDSCs (1 × 10^6^/mL) were seeded in the upper chambers of the inserts 30 min after incubation with culture supernatants of the differential expression Hepa1-6 cells at each concentration. Next recombinant murine CXCL1 (PeproTech, Rocky Hill, NJ, USA) were placed in the lower chamber at a concentration of 0, 10, or 100 ng/mL. The number of MDSCs in the bottom compartment was counted 24 h later.

To evaluate the suppressed migration of MDSCs, MDSCs (1 × 10^6^/mL) were plated in the upper chambers of the inserts 30 min after incubation with the CXCR2 antagonist at a concentration of 0, 100, or 1000 ng/mL at each concentration, the number of MDSCs in the bottom compartment was counted 24 h later. Next recombinant murine CXCL1 (PeproTech, Rocky Hill, NJ, USA) was placed in the lower chamber at a concentration of 10 ng/ml. After incubation for 24 h, the number of MDSCs in the bottom compartment was counted.

### Western blot analyses

Western blotting was performed as previously described^50^. The primary antibodies are used as follows: anti-β-actin (Proteintech, Wuhan, China, 60008-1-Ig), anti-Lamin B1 (Proteintech, 12987-1-AP), anti-SLC7A2 (Abcam, ab140831), anti-p-P65 (Abcam, ab86299), anti-P65 (Abclonal, Wuhan, China, A19653), anti-p50 (Abclonal, A6667), anti-p-IKBα (Abclonal, AP0614), anti-p-AKT (Cell signaling technology, MA, USA, #4060), anti-AKT (Cell signaling technology, #4685), anti-p-JNK (Cell Signaling Technology, #9255), anti-JNK (Cell signaling technology, #9252), anti-p-ERK (Cell signaling technology, #4370), anti-ERK (Cell Signaling Technology, #4695), anti-p-P38 (Cell Signaling Technology, #4511), anti-P38 (Cell Signaling Technology, #8690), anti-p-stat3 (Tyr705) (Cell Signaling Technology, #9145), anti-stat3 (Tyr705) (Cell Signaling Technology, #4904), G9a (EHMT2) (Abcam, ab185050), H3K9me2 (Cell Signaling Technology, #4658), Twist1 (Cell Signaling Technology, #69366), Vimentin (Cell Signaling Technology, #5741), E-cadherin (Cell Signaling Technology, #3195), N-cadherin (Cell Signaling Technology, #13116), MMP9 (Cell Signaling Technology, #13667).

### Quantitative real-time PCR (qRT-PCR)

Total RNA was extracted using TRIzol Reagent (Invitrogen) and reverse transcription was performed within the Advantage RT-for-PCR Kit (Takara) according to the manufacturer’s instructions. The real-time PCR analysis was processed using a SYBR Green PCR Kit (Takara). The cycling parameters were as follows: 95 °C for 30 s, 60 °C for 5 s, and 70 °C for 30 s for 40 cycles. qPCRs were run using SYBR Premix ExTaq (TaKaRa, Otsu, Japan) on ABI StepOne system (Applied Biosystems, Carlsbad, CA, USA). 2^−ΔΔCt^ was used to calculate the fold changes. All reactions were performed in duplicate. The primer sequences are listed in Supplementary Table S4.

### Chromatin immunoprecipitation assay (ChIP)

Briefly, Transfected cells were cross-linked in 1% formaldehyde at 37 °C for 10 min. After washing with PBS, resuspended the cells in 300 μl of lysis buffer, and sonicated to fragment the DNA. Herring sperm DNA (Sigma-Aldrich, USA) and a slurry of Protein G-Sepharose were performed to clear the supernatant. In the presence of Protein G-Sepharose beads and herring sperm DNA, the cleared supernatant was incubated with antibodies (p-P65, H3K9me2) or control IgG for 2 h. Then the PCR was used to amplify the corresponding binding sites on the promoters (Supplementary Table S4 for the primer sequences). The experiments were repeated independently at least three times.

### Luciferase reporter assays

Luciferase activity was detected using the Dual Luciferase Assay (Promega, Madison, WI) according to the manufacturer’s instructions. The lysed cells were centrifuged at maximum speed for 1 min. The relative luciferase activity was measured using ModulusTM TD20/20 Luminometer (Turner Biosystems, USA), and the transfection efficiency was normalized and analyzed according to Renilla luciferase activity.

### Enzyme-linked immunosorbent assay

Tumor tissue was performed in a bead homogenizer, and sonicated in 1× RIPA buffer (Thermo Fisher Scientific) with a protease inhibitor cocktail (MedChemExpress, Monmouth Junction, NJ, USA) and a phosphatase inhibitor cocktail (MedChemExpress, Monmouth Junction, NJ, USA), and was centrifuged at 16,000 × *g* for 10 min at 4 °C. Mouse blood was obtained from tumor-bearing mice and was allowed to clot for 30 min on ice, before being centrifuged at 16,000 × *g* for 10 min at 4 °C and serum was aspirated. Samples were subjected to ELISA analysis. Mouse CXCL1 protein levels were measured by mouse Quantikine ELISA kits for CXCL1 (R&D systems, Minneapolis, MN, USA) according to the manufacturer’s protocols.

Human CXCL1 protein levels in culture supernatants were measured using Human ABTS ELISA Development Kits for CXCL1 (DY275, R&D Systems) according to the manufacturer’s protocols.

### CCK-8 assay

1000 cells were seeded into 96-well plates containing 100 μl of complete medium per well. At a certain time point, CCK-8 solution (Promoter, Hubei, China) and complete medium were mixed at a 1:9 ratio to replace the original medium, and incubated at 37 °C for 2 h. The absorbance of the sample was detected at 450 nm with a microplate reader (Tecan Group, Ltd, Zürich, Switzerland) and each was performed three times.

### Colony formation assay

The transfected cells were seeded in 6-well plates at a density of 1000 cells per well. The cells formed stable colonies after 14–18 days. After fixed with 4% paraformaldehyde and stained with 0.5% crystal violet solution, count more than 50 cells with three replicates in each group.

### In vitro invasion and migration assay

For the migration and invasion assays, a 24-well chamber with 8-μm pore filter (Corning, Inc, NY, USA) was performed. For migration assay, 5 × 10^4^ cells were inoculated in the upper chamber with serum-free medium. For invasion assay, the upper chamber was coated with 200 mg/ml of Matrigel and used after coagulation. Then, 1 × 10^5^ cells were seeded in the upper chamber. The mean values of triplicate assays under each operation condition were applied.

### Datasets

TIMER (http://timer.cistrome.org/), ChIPBase (http://rna.sysu.edu.cn/chipbase/), Ualcan (http://ualcan.path.uab.edu/index.html), GEO (https://www.ncbi.nlm.nih.gov/geo/) and TCGA (https://cancergenome.nih.gov) datasets were used to determine the expression of SLC7A2 and G9a mRNA in human cancer tissues compared to normal specimens.

### Statistical analysis

All values were recorded as the mean ± standard deviation (sd). *P* values were statistically analyzed by the *χ*^2^ test for categorical variables between the two groups and by Student’s test for quantitative data. Parametric One-way analysis of variance (ANOVA) test was used for comparisons between more than two groups. Survival was calculated with the Kaplan–Meier method (log-rank test). Statistical values were calculated with SPSS software (SPSS Inc., Chicago, IL, USA), version 22.0. In all assays, *P* ≤ 0.05 was considered as statistically significant.

## Supplementary information

Supplementary Figure 1

Supplementary Figure 2

Supplementary Figure 3

Supplementary Figure 4

Supplementary Figure 5

Supplementary Figure 6

Supplementary Table S1

Supplementary Table S2

Supplementary Table S3

Supplementary Table S4

Supplementary Table S5

Supplementary Figure Legends

## Data Availability

The data generated and analyzed in this study are available from the corresponding authors upon request.
