# Supplementary figures and images for: SLC7A2 deficiency promotes hepatocellular carcinoma progression by enhancing recruitment of myeloid-derived suppressors cells
Source: Cell Death Dis. 2021 Jun 2;12(6):570. doi: 10.1038/s41419-021-03853-y (PMC8190073; doi:10.1038/s41419-021-03853-y)

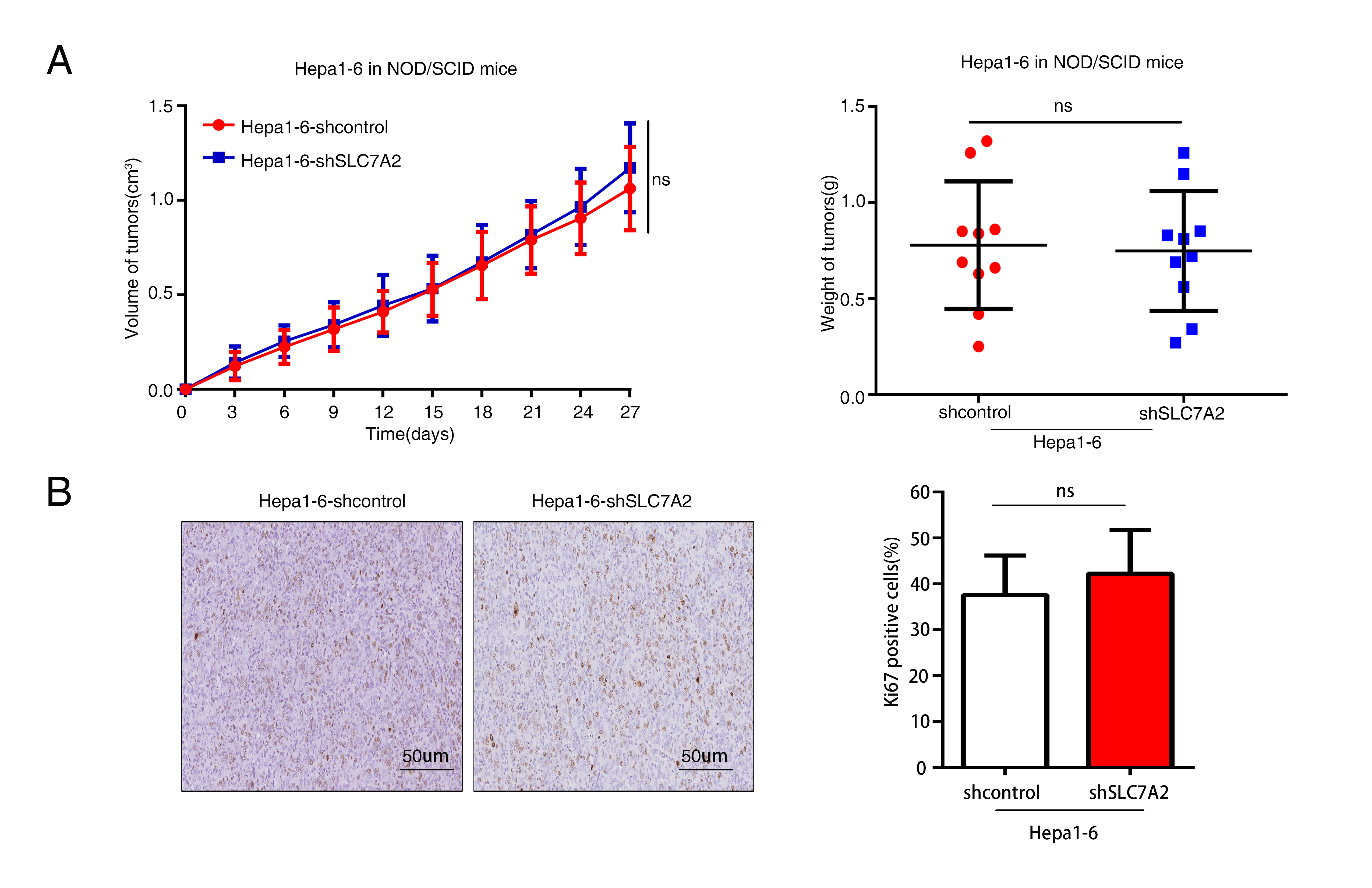

Supplement: Supplementary file 3 — Supplementary Figure 3 [file 41419_2021_3853_MOESM3_ESM.tif]

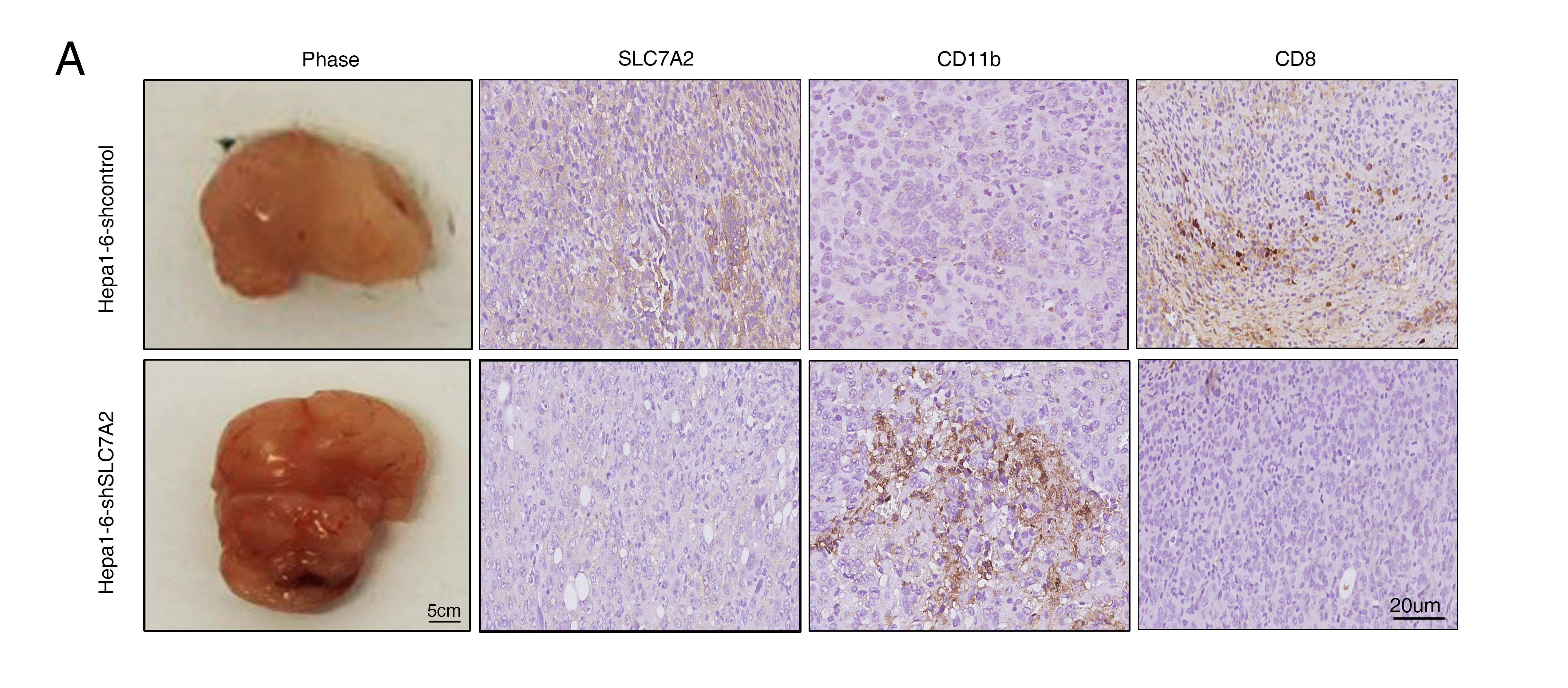

Supplement: Supplementary file 4 — Supplementary Figure 4 [file 41419_2021_3853_MOESM4_ESM.tif]

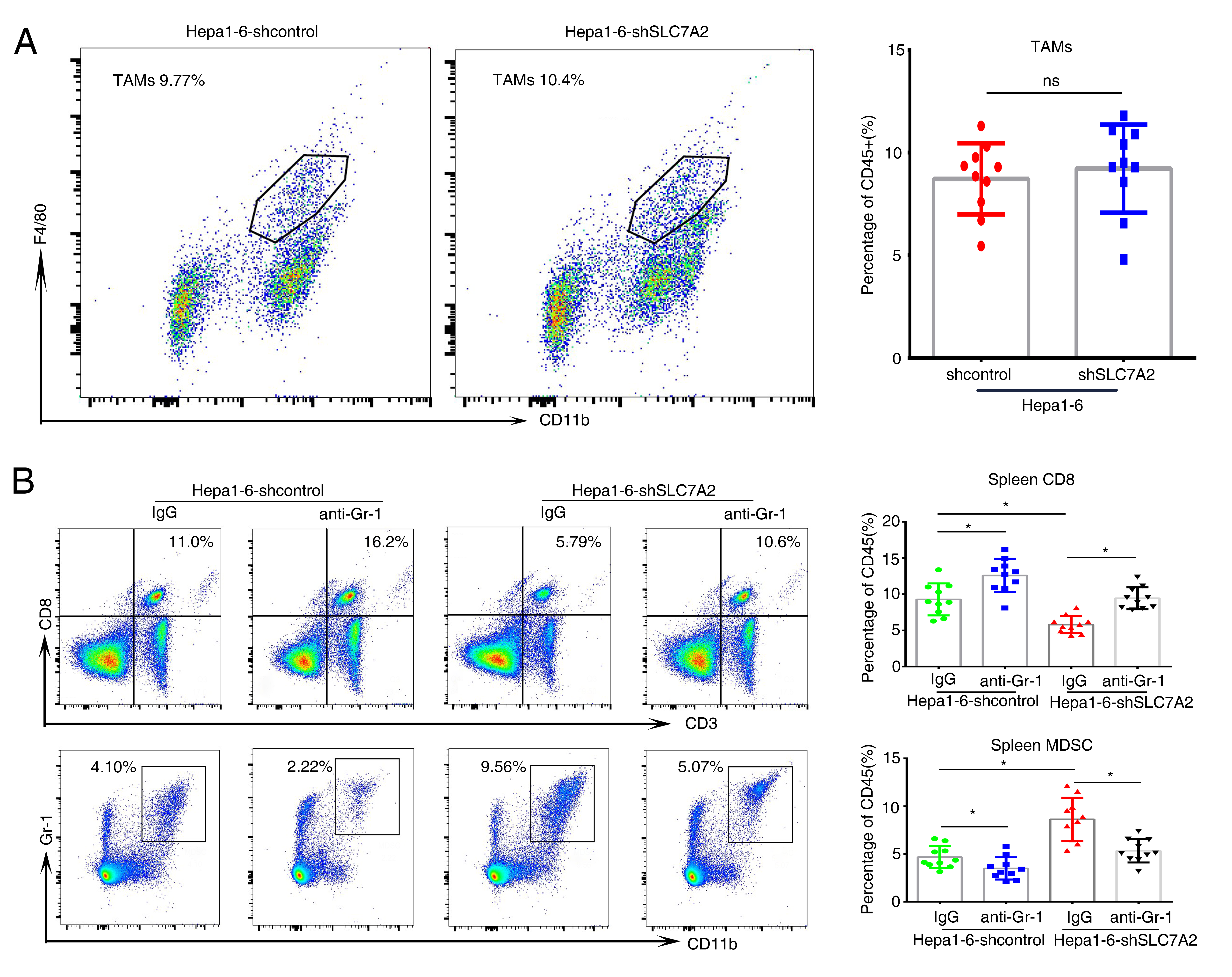

Supplement: Supplementary file 5 — Supplementary Figure 5 [file 41419_2021_3853_MOESM5_ESM.tif]
