# Supplementary material for: SLC7A2 deficiency promotes hepatocellular carcinoma progression by enhancing recruitment of myeloid-derived suppressors cells
Source: Cell Death Dis. 2021 Jun 2;12(6):570. doi: 10.1038/s41419-021-03853-y (PMC8190073; doi:10.1038/s41419-021-03853-y)
Supplement: Supplementary file 7 — Supplementary Table S1 [file 41419_2021_3853_MOESM7_ESM.docx]

Supplementary Table 1 List of genes differentially expressed in Huh7 cells after SLC7A2 knockdown using a human Chemokines and Receptors PCR array

| Gene | Huh7-shSLC7A2(fold change) | Function |
| --- | --- | --- |

CXCL1 5.63 Chemokine (C-X-C motif) Ligands

| CXCL2 | 4.42 | Chemokine (C-X-C motif) Ligands | |
| --- | --- | --- | --- |
| CXCL9 | 4.21 | Chemokine (C-X-C motif) Ligands | |
| CXCL10 | 4.04 | Chemokine (C-X-C motif) Ligands | |
| VHL | 3.89 | Other Chemokines and Related Genes | |
| HIF1A | 3.55 | Other Chemokines and Related Genes | |
| IL18 | 2.78 | Other Chemokines and Related Genes | |
| IL8RA(CXCR1) | 2.56 | Other Chemokines and Related Genes | |
| IL8 | 2.03 | Other Chemokines and Related Genes | |
| SDF2 | 2.03 | Other Chemokines and Related Genes | |
| CCL17 | 2.02 | Chemokine (C-C motif) Ligands | |
| CCRL2 | 2.01 | Chemokine (C-C motif) Receptors | |
| MMP7 | 1.98 | Other Chemokines and Related Genes | |
| CCL8 | 1.98 | Chemokine (C -C motif) Ligands | |
| GPR31 | 1.96 | Other Chemokines and Related Genes | |
| CXCR4 | 1.95 | Chemokine (C-X-C motif) Receptors | |
| NFKB1 | 1.91 | Other Chemokines and Related Genes | |
| CCRL1 | 1.86 | Chemokine (C-C motif) Receptors | |
| MYD88 | 1.81 | Other Chemokines and Related Genes | |
| CCR2 | 1.81 | Chemokine (C-C motif) Receptors | |
| CXCL3 | 1.77 | Chemokine (C-X-C motif) Ligands | |
| CCR7 | 1.74 | Chemokine (C-C motif) Receptors | |
| CKLFSF2 | 1.74 | Other Chemokines and Related Genes | |
| CKLFSF3 | 1.72 | Other Chemokines and Related Genes | |
| CCR10 | 1.69 | Chemokine (C-C motif) Receptors | |
| CSF3 | 1.69 | Other Chemokines and Related Genes | |
| CCL2 | 1.67 | Chemokine (C-C motif) Ligands |  |
| CCL3 | 1.66 | Chemokine (C-C motif) Ligands | |
| CCL5 | 1.66 | Chemokine (C-C motif) Ligands | |
| CKLFSF1 | 1.54 | Other Chemokines and Related Genes | |
| TLR4 | 1.46 | Other Chemokines and Related Genes | |
| CCL18 | 1.36 | Chemokine (C-C motif) Ligands | |
| CCL19 | 1.28 | Chemokine (C-C motif) Ligands | |
| CX3CR1 | 1.22 | Other Chemokines and Related Genes | |
| CCL11 | 1.23 | Chemokine (C-C motif) Ligands | |
| CX3CL1 | 1.19 | Other Chemokines and Related Genes | |
| MMP2 | 1.03 | Other Chemokines and Related Genes | |
| TNFSF14 | 1.01 | Other Chemokines and Related Genes | |
| CXCL5 | 1.01 | Chemokine (C-X-C motif) Ligands |  |
| CXCL6 | -1.01 | Chemokine (C-X-C motif) Ligands | |
| CCR6 | -1.08 | Chemokine (C-C motif) Receptors | |
| CCR4 | -1.13 | Chemokine (C-C motif) Receptors | |
| SLIT2 | -1.16 | Other Chemokines and Related Genes | |
| CCR3 | -1.18 | Chemokine (C-C motif) Receptors | |
| CXCR6 | -1.21 | Chemokine (C-X-C motif) Receptors | |
| CXCL13 | -1.25 | Chemokine (C-X-C motif) Ligands | |
| CCR5 | -1.26 | Chemokine (C-C motif) Receptors | |
| IL16 | -1.35 | Other Chemokines and Related Genes | |
| GPR81 | -1.37 | Other Chemokines and Related Genes | |
| GPR77 | -1.38 | Other Chemokines and Related Genes | |
| C5R1 | -1.41 | Other Chemokines and Related Genes | |
| CKLFSF4 | -1.49 | Other Chemokines and Related Genes | |
| CXCL11 | -1.49 | Chemokine (C-X-C motif) Ligands | |
| CCL7 | -1.50 | Chemokine (C-C motif) Ligands | |
| SCYE1 | -1.52 | Other Chemokines and Related Genes | |
| GDF5 | -1.53 | Other Chemokines and Related Genes | |
| TREM1 | -1.54 | Other Chemokines and Related Genes | |
| AGTRL1 | -1.55 | Other Chemokines and Related Genes | |
| CCR8 | -1.64 | Chemokine (C-C motif) Receptors | |
| ECGF1 | -1.68 | Other Chemokines and Related Genes | |
| XCR1 | -1.73 | Other Chemokines and Related Genes | |
| CCL16 | -1.45 | Chemokine (C-C motif) Ligands | |
| CXCR3 | -1.54 | Chemokine (C-X-C motif) Receptors | |
| IL13 | -1.57 | Other Chemokines and Related Genes | |
| CCL1 | -1.62 | Chemokine (C-C motif) Ligands | |
| CYFIP2 | -1.66 | Chemokine (C-X-C motif) Receptors | |
| BDNF | -1.72 | Other Chemokines and Related Genes | |
| CCBP2 | -1.81 | Other Chemokines and Related Genes | |
| IL1A | -1.87 | Other Chemokines and Related Genes | |
| CMKLR1 | -1.89 | Other Chemokines and Related Genes | |
| XCL1 | -1.90 | Other Chemokines and Related Genes | |
| CCL4 | -1.91 | Chemokine (C-C motif) Ligands | |
| CXCL12 | -1.95 | Chemokine (C-X-C motif) Ligands | |
| TLR2 | -1.97 | Other Chemokines and Related Genes | |
| CCL15 | -1.98 | Chemokine (C-C motif) Ligands | |
| CKLF | -1.98 | Other Chemokines and Related Genes | |
| TCP10 | -2.01 | Other Chemokines and Related Genes | |
| CCR1 | -2.03 | Chemokine (C-C motif) Receptors | |
| LTB4R | -2.06 | Other Chemokines and Related Genes | |
| C5 | -2.08 | Other Chemokines and Related Genes | |
| CCL13 | -2.19 | Chemokine (C-C motif) Ligands | |
| IL4 | -2.16 | Other Chemokines and Related Genes | |
| TNF | -2.17 | Other Chemokines and Related Genes | |
