# Supplementary material for: SLC7A2 deficiency promotes hepatocellular carcinoma progression by enhancing recruitment of myeloid-derived suppressors cells
Source: Cell Death Dis. 2021 Jun 2;12(6):570. doi: 10.1038/s41419-021-03853-y (PMC8190073; doi:10.1038/s41419-021-03853-y)
Supplement: Supplementary file 8 — Supplementary Table S2 [file 41419_2021_3853_MOESM8_ESM.docx]

Supplementary Table S2. Correlation Between CD11b Expression and Clinicopathological Characteristics in HCC patients.

| Clinicopathological variables | | Tumor CD11b expression | | *P* Value |  |
| --- | --- | --- | --- | --- | --- |
|  |  | Negative (n=61) | Positive (n=25) |  |  |
| Age(years) <55 | | 34 | 15 | 0.812 |  |
|  | ≥55 | 27 | 10 |  |  |
| Sex | female | 14 | 5 | 1.000 |  |
|  | male | 47 | 20 |  |  |
| Serum AFP | ≤20ng/ml | 12 | 3 | 0.537 |  |
|  | >20ng/ml | 49 | 22 |  |  |
| Cirrrhosis | absent | 11 | 8 | 0.749 |  |
|  | present | 50 | 3 |  |  |
| Child-pugh score | Class A | 52 | 20 | 0.537 |  |
|  | Class B | 9 | 5 |  |  |
| Tumor number | single | 44 | 21 | 0.284 |  |
|  | multiple | 17 | 4 |  |  |
| Maximal tumor size | ≤5cm | 40 | 13 | 0.329 |  |
|  | >5cm | 21 | 15 |  |  |
| Tumor encapsulation | absent | 22 | 10 | 0.056 |  |
|  | present | 39 | 24 |  |  |
| Microvascular invasion | absent | 33 | 6 | 0.016* |  |
|  | present | 28 | 19 |  |  |
| TNM stage | I-II | 46 | 14 | 0.119 |  |
|  | III | 15 | 11 |  |  |
