# Supplementary material for: SLC7A2 deficiency promotes hepatocellular carcinoma progression by enhancing recruitment of myeloid-derived suppressors cells
Source: Cell Death Dis. 2021 Jun 2;12(6):570. doi: 10.1038/s41419-021-03853-y (PMC8190073; doi:10.1038/s41419-021-03853-y)
Supplement: Supplementary file 9 — Supplementary Table S3 [file 41419_2021_3853_MOESM9_ESM.docx]

Supplementary Table S3. Correlation Between G9a Expression and Clinicopathological Characteristics in HCC patients.

| Clinicopathological variables | | Tumor G9a expression | | *P* Value |  |
| --- | --- | --- | --- | --- | --- |
|  |  | Negative (n=35) | Positive (n=51) |  |  |
| Age(years) <55 | | 21 | 28 | 0.664 |  |
|  | ≥55 | 14 | 23 |  |  |
| Sex | female | 10 | 9 | 0.292 |  |
|  | male | 25 | 42 |  |  |
| Serum AFP | ≤20ng/ml | 8 | 7 | 0.386 |  |
|  | >20ng/ml | 27 | 44 |  |  |
| Cirrrhosis | absent | 6 | 8 | 1.000 |  |
|  | present | 29 | 43 |  |  |
| Child-pugh score | Class A | 31 | 41 | 0.383 |  |
|  | Class B | 4 | 10 |  |  |
| Tumor number | single | 21 | 44 | 0.010* |  |
|  | multiple | 14 | 7 |  |  |
| Maximal tumor size | ≤5cm | 25 | 28 | 0.176 |  |
|  | >5cm | 10 | 23 |  |  |
| Tumor encapsulation | absent | 11 | 26 | 0.081 |  |
|  | present | 24 | 25 |  |  |
| Microvascular invasion | absent | 18 | 21 | 0.384 |  |
|  | present | 17 | 30 |  |  |
| TNM stage | I-II | 27 | 33 | 0.242 |  |
|  | III | 8 | 18 |  |  |
