# Supplementary material for: SLC7A2 deficiency promotes hepatocellular carcinoma progression by enhancing recruitment of myeloid-derived suppressors cells
Source: Cell Death Dis. 2021 Jun 2;12(6):570. doi: 10.1038/s41419-021-03853-y (PMC8190073; doi:10.1038/s41419-021-03853-y)
Supplement: Supplementary file 10 — Supplementary Table S4 [file 41419_2021_3853_MOESM10_ESM.docx]

Supplementary Table S4. Primer sequences used in the study

| Primer name | | Primer sequences | | Enzyme | |
| --- | --- | --- | --- | --- | --- |
| Primers for real-time PCR: | |  | |  | |
| Human-SLC7A2 sense: | | 5'- TGCAGGGGTCATTTCTGCTT-3' | |  | |
| Human-SLC7A2 antisense: | | 5'-GGGCGAAGGGTAGCGATTC-3' | |  | |
| Human-CXCL1 sense: | | 5′-GCAGGGAATTCACCCCAAGA-3′ | |  | |
| Human-CXCL1 antisense: | | 5′-GGTAGCCCTTGTTTCCCCC-3′ | |  | |
| Mouse-CXCL1 sense: | | 5’-AAGACAAGCTTCCATCCCCG-3’ | |  | |
| Mouse-CXCL1 antisense: | | 5’-CGGAGTACAGGGTGTTTCCC-3’ | |  | |
| Human-β-actin sense: | | 5’-CATGTACGTTGCTATCCAGGC -3’ | |  | |
| Human-β-actin antisense: | | 5’-CTCCTTAATGTCACGCACGAT -3’ | |  | |
| Mouse-β-actin sense: | | 5′-CATCTCACCTGAAGCACCCT-3′ | |  | |
| Mouse-β-actin antisense: | | 5′-CGGAGTCCATCACAATGCCT-3′ | |  | |
| Primers for CXCL1 promoter construct: | | | |  | |
| (-1443/+127) CXCL1 sense: | | 5’-TATAGGTACCAATCTGCAGGCGATGCTT-3’ | | KpnI | |
| (-817/+127) CXCL1 sense: | | 5’-TATAGGTACCACCTGAACCCCTCCTACA-3’ | | KpnI | |
| (-366/+127) CXCL1 sense: | | 5’-TATAGGTACCTTCTCTGTGGTGGTTCTC-3’ | | KpnI | |
| (-71/+127) CXCL1 sense: | | 5’-TATAGGTACCATCGATCTGGAACTCCGG-3’ | | KpnI | |
| Antisense: | | 5’-ATATAAGCTTGCAATCCCCGGCTCCTGC-3’ | | HindIII | |
| Primers for CXCL1 promoter site-directed mutagenesis:  p65 binding site: | | | |  | |
| binding site 3 mutation sense: | | 5’-CCCTCCTACAGatcgAAGATTTCCAG-3’ | |  | |
| binding site 3 mutation antisense: | | 5’-CTGGAAATCTTcgatCTGTAGGAGGG-3’ | |  | |
| binding site 2 mutation sense: | | 5’-GGTGGTTCTCAtaacTCCGCCCCAGC -3’ | |  | |
| binding site 2 mutation antisense: | | 5’-GCTGGGGCGGAgttaTGAGAACCACC -3’ | |  | |
| binding site 1 mutation sense: | | 5’-GGAACTCCGGGcgcgTCCCTGGCCCG -3’ | |  | |
| binding site 1 mutation antisense: | | 5’-CGGGCCAGGGAcgcgCCCGGAGTTCC -3’ | |  | |
| Primers used for ChIP in the CXCL1 promoter: | | | |  | |
| distant region sense: | | 5’-TGCCAGTGCTTGCAGACC -3’ | |  | |
| distant region antisense: | | 5’-CCTGCATCCCCCATAGTT -3’ | |  | |
| binding site 3 sense: | | 5’-TTCTAGATCAAACCTGAA -3’ | |  | |
| binding site 3 antisense: | | 5’-CAGGGGATTTTGAAAGTT -3’ | |  | |
| binding site 2 sense: | | 5’-GGTCGCGCCTTCTCTGTG -3’ | |  | |
| binding site 2 antisense: | | 5’-AGCTCCTTCTCCGTTCCC -3’ | |  | |
| binding site 1 sense: | | 5’-TCGGGATCGATCTGGAAC -3’ | |  | |
| binding site 1 antisense: | | 5’-CGGGGGCTCCGGGCTTTC -3’ | |  | |
| Primers sequences for ChIP-qPCR:  ChIP-SLC7A2 sense: 5’-GACTCCTAAAGTAAACAA -3’  ChIP-SLC7A2 antisense: 5’-CACCTGGGCTTCCATTTAT -3’ | | | |  |  |
