# Supplementary material for: SLC7A2 deficiency promotes hepatocellular carcinoma progression by enhancing recruitment of myeloid-derived suppressors cells
Source: Cell Death Dis. 2021 Jun 2;12(6):570. doi: 10.1038/s41419-021-03853-y (PMC8190073; doi:10.1038/s41419-021-03853-y)
Supplement: Supplementary file 11 — Supplementary Table S5 [file 41419_2021_3853_MOESM11_ESM.docx]

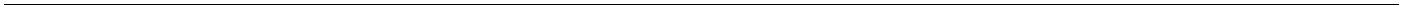

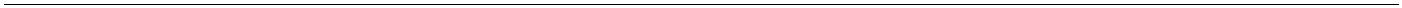
Supplementary Table S5. Knockdown shRNA sequences used in this study

TRC number Sequence

Human:

ShSLC7A2 CCGGAGTCTAGAAGACACCAAATTACTCGAGTAATTTGGTGTCTTCTAGACTTTTTTG

Mouse:


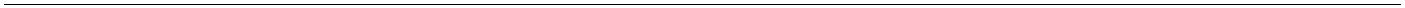
ShSLC7A2 CCGGCGTCCTTACTTGTCTGCTTTACTCGAGTAAAGCAGACAAGTAAGGACGTTTTTG
