# Supplementary material for: SLC7A2 deficiency promotes hepatocellular carcinoma progression by enhancing recruitment of myeloid-derived suppressors cells
Source: Cell Death Dis. 2021 Jun 2;12(6):570. doi: 10.1038/s41419-021-03853-y (PMC8190073; doi:10.1038/s41419-021-03853-y)
Supplement: Supplementary file 12 — Supplementary Figure Legends [file 41419_2021_3853_MOESM12_ESM.docx]

**Supplementary Figure Legends**

**Figure S1.** Data from The Cancer Genome Atlas dataset showing relative mRNA expression levels of SLC7A2 in multiple types of tumor.

(A, B) Representative data obtained from the GEPIA and UALCAN dataset showing relative mRNA expression levels of SLC7A2 in multiple types of tumor.

The data are shown as the mean ± SD, **P* < 0.05.

**Figure S2.** (A) Western blotting analyzed performed the expression of SLC7A2 in MHCC97H、Huh7、H22 and Hepa1-6. (B) Western blotting analyzed performed the expression of SLC7A2 in H22 and Hepa1-6 cells after lentivirus transfection. (C) The effects of SLC7A2 on mice HCC cell proliferation were measured by a CCK-8 assay. (D) Transwell assay shown the abilities of migration and invasion in the indicated HCC cells. The scale bar represents 100 μm. (E) Transwell assay shown the abilities of migration and invasion in the indicated mice HCC cells. (F) FACS analysis of the expression of SLC7A2 in Huh7 and MHCC97H cells after lentivirus transfection labeled with Annexin-V FITC and propidium iodide (PI) as markers for apoptosis. (G) Western blotting analyzed performed the expression of SLC7A2 in Huh7 and MHCC97H cells after lentivirus transfection. The scale bar represents 100 μm. Data are the mean ± SEM from triplicate experiments. *P < 0.05 and ***P < 0.001.

**Figure S3.** (A) Growth curves and weight curves of tumors in NOD/SCID mice (n=10 mice per group) in subcutaneously model. (B) IHC staining for Ki67 in the indicated tumors. The data are shown as the mean ± SD, **P* < 0.05.

**Figure S4.** (A) Representative IHC staining images for SLC7A2, CD11b, CD8 in mice tumors tissues.

**Figure S5.** (A) Flow cytometric analysis of tumor-associated macrophages (TAMs) in subcutaneous Hepa1-6-shcontrol and Hepa1-6-shSLC7A2 tumors from immunocompetent mice. n=10. (B) The fraction of CD8^+^ T cells and MDSCs were analyzed by flow cytometry in mouse spleen of subcutaneous tumors treated with anti-Gr-1 antibody or IgG at day 27. CD8^+^ T cells (upper) and MDSCs (lower) (n=10 mice per group). The data are shown as the mean ± SD from the at least three independent experiments; *P < 0.05.

**Figure S6.** (A-C) Multiple bioinformatics databases revealed the relationships between SLC7A2 and DNMT1, DNMT3B, EZH2, EHMT2, HDAC1 and HDAC2.
